# Biologically Active Benzimidazole Hybrids as Cancer Therapeutics: Recent Advances

**DOI:** 10.3390/ph18101454

**Published:** 2025-09-28

**Authors:** Mohamed A. S. Badawy, Stefan Bräse, Taha F. S. Ali, Mohamed Abdel-Aziz, Hamdy M. Abdel-Rahman

**Affiliations:** 1 Department of Pharmaceutical Chemistry, Faculty of Pharmacy, Merit University (MUE), Sohag 82755, Egypt; 2 Institute of Biological and Chemical Systems—Functional Molecular Systems (IBCS-FMS), Karlsruhe Institute of Technology (KIT), Kaiserstrasse 12, 76131 Karlsruhe, Germany; 3 Institute of Organic Chemistry (IOC), Karlsruhe Institute of Technology (KIT), Kaiserstrasse 12, 76131 Karlsruhe, Germany; 4 Department of Medicinal Chemistry, Faculty of Pharmacy, Minia University, Minia 61519, Egypt; taha.ali@mu.edu.eg (T.F.S.A.); abulnil@mu.edu.eg (M.A.-A.); 5 Department of Medicinal Chemistry, Faculty of Pharmacy, Assiut University, Assiut 71526, Egypt; hamdym@aun.edu.eg; 6 Department of Pharmaceutical Chemistry, Faculty of Pharmacy, Badr University in Assiut (BUA), Assiut 71111, Egypt

**Keywords:** benzimidazole derivatives, anticancer agents, docking, target proteins, mechanisms of action, structure–activity relationship

## Abstract

Cancer is a highly significant medical concern, as it is the second most prevalent cause of mortality after cardiovascular diseases. It arises due to dysregulated cell cycle control, leading to a gradual decline in cellular differentiation and unrestricted cellular proliferation. Therefore, the primary objective for researchers is to develop a cancer treatment that addresses drug resistance while providing effective therapeutic benefits and minimizing side effects. Benzimidazole has garnered significant attention because it serves as an auxiliary isostere of nucleotides, which are found in several natural and biologically active molecules. Benzimidazole compounds possess a privileged pharmacophore that exhibits various pharmacological actions. Several benzimidazole derivatives exhibit dual or multiple anticancer properties through diverse mechanisms, focusing on specific compounds or employing strategies that are not gene specific. Furthermore, many drugs based on benzimidazole have previously been approved to treat cancer. This comprehensive review encompasses the most important reports on various benzimidazole hybrids, highlighting their anticancer significance, mechanism of action, and structure-activity relationships from 2005 up to 2025. These provide valuable knowledge for designing effective anticancer drugs.

## 1. Introduction

### 1.1. Cancer

The term ‘cancer’ originates from the Latin word, which was previously referred to as ‘karkinoma’ in Greek [1]. Cancer was initially identified as a proliferating tissue mass, referred to as a tumor. They could examine its appearance, growth rate, and the frequency with which it seemed to take a “bite” out of the body [2,3]. Many definitions of cancer align with the National Cancer Institute’s definition: “Cancer is a disease in which some of the body’s cells grow uncontrollably and spread to other parts of the body.” [4,5]. Cancer has emerged as a highly significant medical issue because of its incidence, being the second leading cause of death, and the increasing mortality rate around the globe [6]. The most recent GLOBOCAN analysis indicates that there were 18,094,716 new cancer cases globally, comprising 51.63% males and 48.36% females, along with 9,894,402 cancer-related deaths, with 55.49% males and 44.51% females [7]. Cancer is complex, and cancer management is difficult because of cancer recurrence, drug toxicity, drug resistance, tumor formation, and the limited efficacy of drug research in progressing to clinical trials [8]. Thus, it is critically important to design and synthesize new, safe, and effective anticancer compounds that can overcome this resistance. Recent discoveries in anticancer drugs have highlighted the significance of various benzimidazole derivatives, which are noted for their diverse biological activities and potential applications in cancer treatment. The distinctive core structure of benzimidazole, along with its low toxicity, positions it as an advantageous scaffold in the development of anticancer drugs [9,10].

### 1.2. Benzimidazole

**Figure 1:**
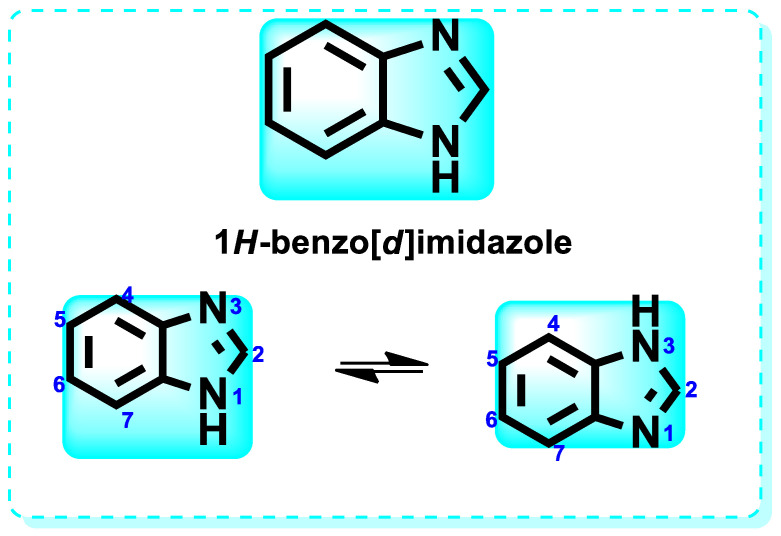
Structure of *1H*-Benzo[d]imidazole.

**Scheme 1:**
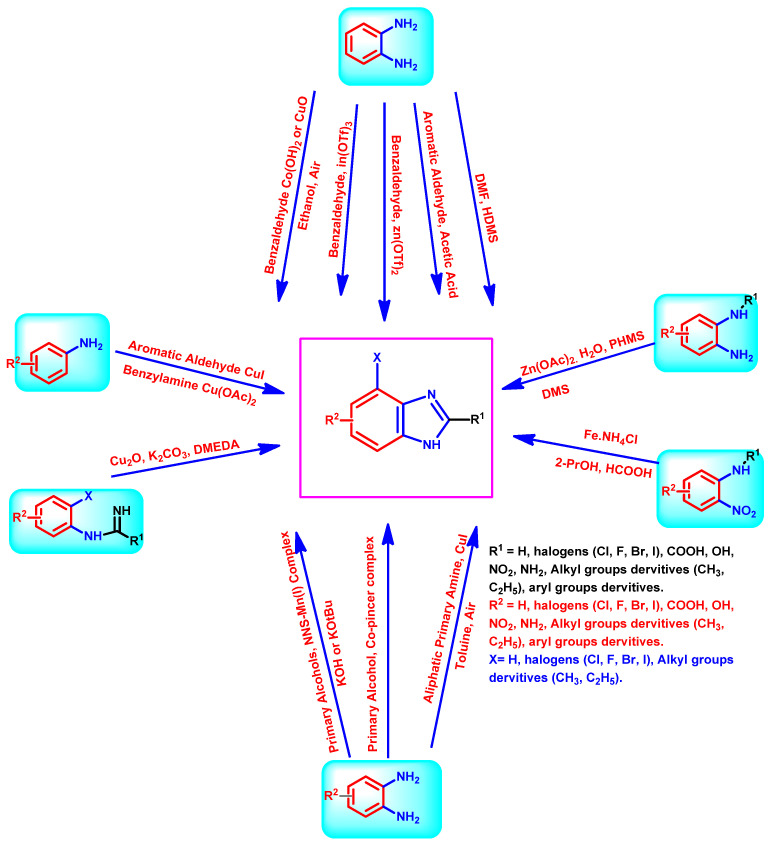
Diverse synthesis pathways for the benzimidazole ring.

Benzimidazole is a compound consisting of an imidazole ring with five members fused to a benzene ring with six members at positions 4 and 5 [11]. The compound is known by its IUPAC designation, *1H*-benzimidazole, and is sometimes referred to as *1H*-1,3-benzimidazole or *1H*-benzo[d]imidazole. It is alternatively referred to as benziminazole, azindole, 1,3-diazaindene, 3-benzodiazole, 3-azaindole, or benzoglyoxaline. It has a melting point range of 170–172 °C and is found in the form of white crystals [12]. The planar benzimidazole ring exhibits tautomerization of the hydrogen atom between the double-bonded nitrogen and the single-bonded nitrogen Figure 1. If N-substituted derivatives impede tautomerization, they yield two isolated isomers. The NH group of benzimidazole exhibits high acidity and mild basicity, and it is also capable of forming salts [13]. Currently, numerous researchers have described various methods for synthesizing 1-substituted or 1,2-disubstituted benzimidazole derivatives using distinct moieties under diverse reaction conditions. The primary reaction of monosubstituted benzimidazoles (MSBs) and disubstituted benzimidazoles (DSBs) involves the condensation of o-phenylenediamine with the ketonic group of aliphatic or aromatic benzaldehyde, utilizing various bases, catalysts, and solvent conditions, as detailed in Scheme 1 [13]. Hobrecker first prepared it in 1872 [14]. The most common benzimidazole molecule in nature is N-ribosyl-dimethyl benzimidazole, which is crucial for cobalt in the structure of vitamin B12 [15]. Over the years, the privileged pharmacophore of benzimidazole has made it the preferred skeleton in drug designing and discovery [16,17].

### 1.3. Biological Activity and Drugs Containing Benzimidazole

The various activities seen may arise among the different substituents attached to the benzimidazole scaffold [18,19,20,21] and allow it to have a wide range of biological activities, like anti-inflammatory [22,23], antifungal [24], proton pump inhibitor [25], antimicrobial [26,27,28,29], anthelmintic [30,31], anticoagulant [32], antiviral [33,34], antihypertensive [35], antiulcer activity [36], antihistamine [37], anticancer [29,38,39], and so on. Table 1 displays several benzimidazole-based medications currently available on the market. Benzimidazoles, initially developed for antiparasitic applications, transitioned into oncology during late-20th-century preclinical studies that demonstrated their ability to disrupt microtubules and induce apoptosis. This was followed by case reports and small trials in the early 2000s, particularly involving albendazole and mebendazole, which catalyzed continued research into their repurposing [40,41]. Furthermore, it is fascinating to note that several anthelmintic benzimidazole medications have been repurposed as anticancer treatments. Mebendazole (MBZ), oxibendazole (OBZ), carbendazim (CBZ), albendazole (ABZ), and fenbendazole (FBZ) are among the examples [41]. Recently, compounds exhibiting anticancer properties that contain the benzimidazole structure have been incorporated into the research focus [42,43,44]. Veliparib, nocodazole, pracinostat, selumetinib, galeterone, glasdegib, bendamustine, dovitinib, and crenolanib are licensed drugs for the treatment of various malignancies [45]. The well-known pathways of anticancer drugs can be categorized into three main groups. The first is the inhibition of enzymes, angiogenesis, DNA synthesis, and microtubules. The second one is gene and transcription regulation. The third is DNA intercalation and groove binding [46]. Targeting the cause of cancer is a widely employed approach for cancer treatment [47,48]. The primary targets for anticancer treatment are topoisomerases, protein kinases, cysteine amidases, and tubulin polymerases [49]. Although multiple methods are already being used, there remains an urgent need to develop innovative treatment techniques that ensure safety and effectiveness against this potentially fatal illness [50]. In this review, we are addressing many current compounds containing benzimidazole that have been evaluated for cancer treatment up to 2025.

## 2. Benzimidazole Targets and SAR as Anticancer Therapy

**Figure 2:**
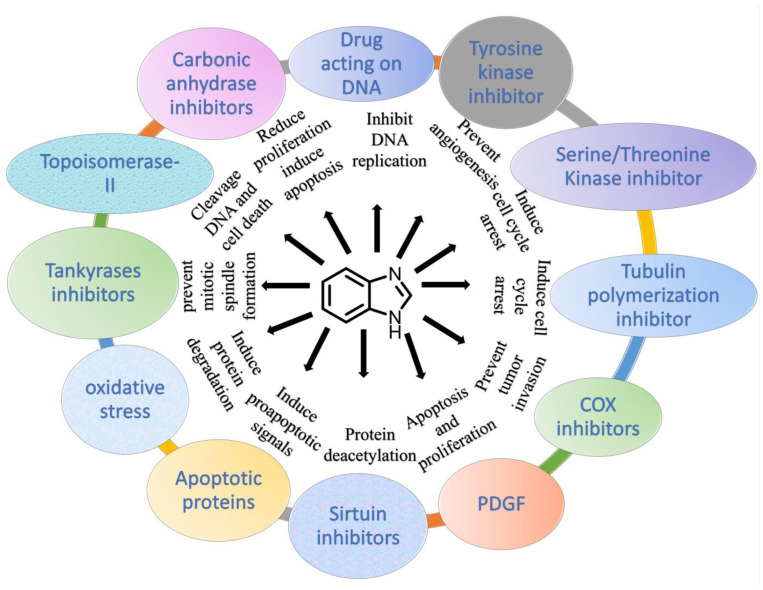
Demonstration of benzimidazole derivatives as anticancer agents.

Primary anticancer therapeutic targets are demonstrated in Figure 2, and the corresponding articles support the effectiveness of benzimidazole compounds as potential anticancer medicines through the development, evaluation, in vivo, and in vitro experiments.

### 2.1. Protein Kinase Inhibitors

**Figure 3:**
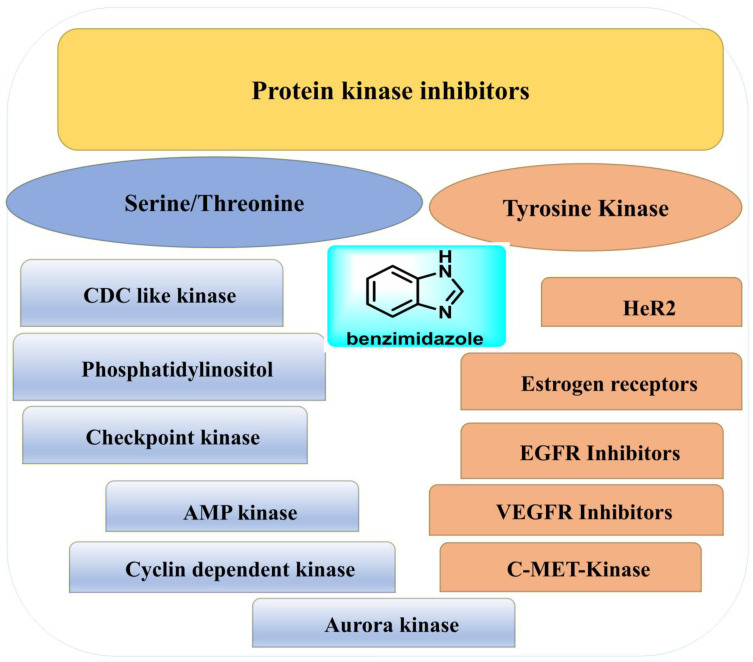
Demonstration of benzimidazoles’ action as protein kinase inhibitors.

Kinases are essential for enabling the passage of signals within cells; therefore, they regulate crucial biological processes, including development, proliferation, differentiation, and normal cell death, as well as various molecular activities [51]. Within the context of live cells, kinases may be continuously activated if there is a problem with the phosphorylation process of the kinase protein, thereby facilitating the advancement of cancer and the growth of tumors [52]. The human genome comprises approximately 535 kinases, categorized into tyrosine and serine/threonine kinases based on their inhibitory activity towards specific protein kinases [53]. Kinase inhibitor synthesis has made considerable use of the benzimidazole scaffold as a structural framework (Figure 3) [54].

#### 2.1.1. Serine/Threonine

Serine/threonine kinases, a subfamily of protein kinases (PK), phosphorylate various proteins that contain residues of the amino acids serine and threonine [55]. It fulfills a crucial role in the regular advancement of the cell cycle, which deviates from normalcy in the context of cancer [56]. CDKs, phosphatidylinositol BRAF/MEK/ERK, and PI3K/AKT/mTOR are examples of serine/threonine kinases [57]. Serine/threonine kinase protein subfamily members are overexpressed in various forms of tumors. As a result, they can be successfully targeted as a therapeutic target for cancer drugs in anticancer therapies [58].

In 2018, the pharmaceutical medication Binimetinib, developed by Array BioPharma, obtained FDA (Food and Drug Administration) approval for commercialization [59]. Binimetinib is predominantly employed to treat melanoma caused by the genetic mutation BRAF (V-raf murine sarcoma viral oncogene homolog B1) [60]. The investigational drug is presently undergoing clinical investigation (phase III) to evaluate its potency in treating cancers in the peritoneal, fallopian tube, colorectal, and ovarian regions [61]. In the context of clinical practice, while dealing with individuals who are suffering from a mutation in the BRAF gene in melanoma, the administration of encorafenib in conjunction with binimetinib has demonstrated favorable effectiveness and safety outcomes when compared to Vemurafenib usage as a standalone therapy [62]. Furthermore, the patients’ group who received a combination of encorafenib treatment and another intervention had a significantly prolonged period of progression-free survival compared to those who had just received encorafenib treatment [63].

##### CDC-like Kinase

Cell division cycle-like kinases (CLKs) are essential components of the splicing machinery responsible for precisely selecting exons [64]. Inhibitors targeting CLKs have been shown to impede cellular proliferation and trigger programmed cell death (apoptosis) by regulating the splicing patterns of genes crucial for cellular proliferation and maintaining their life [65]. Humanity has 4 CDC-like kinase genes, namely CLK1, CLK2, CLK3, and CLK4 [66]. The expression of the CDC-like kinase gene two has significantly increased in breast cancers. Furthermore, it has been observed that the downregulation of this gene efficiently inhibits the tumor cells’ proliferation [67].

**Figure 4:**
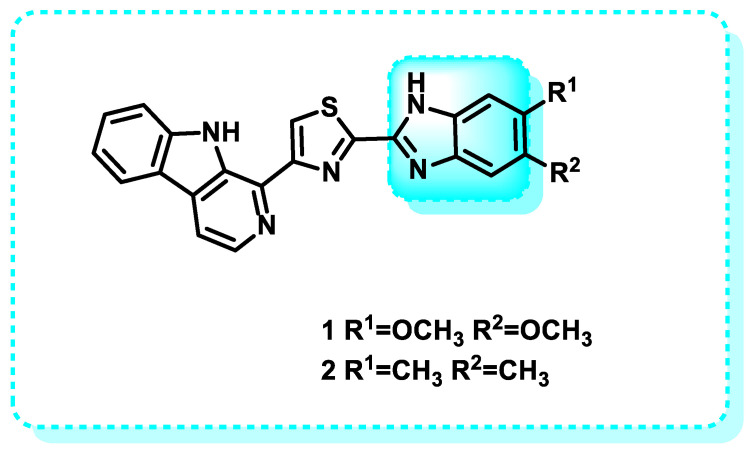
Benzimidazole scaffold in CDC-like kinase inhibitors.

**Figure 5:**
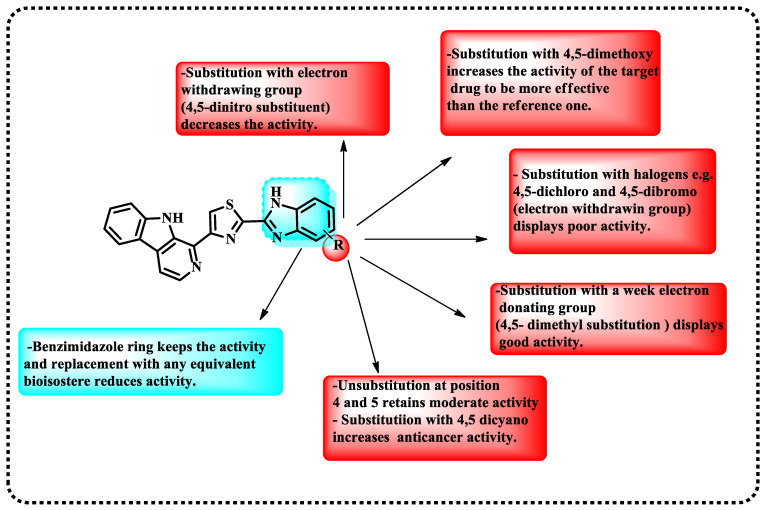
Demonstration of SAR for the synthesized targets.

A novel set of β-carbolines connected by benzimidazole/benzoxazole linkages was developed by integrating effective anticancer moieties. Compounds undergo anticancer evaluation against cell lines derived from human cancer cells, specifically A2780 (ovary), A549 (lung), Colo-205 (colon), and MCF-7 (breast), utilizing the established MTT assay. The hybrid β-carbolines showed a notable and substantial increase in their anticancer effectiveness compared to the reference standard etoposide. Among the hybrid β-carbolines, compounds **1** and **2** (Figure 4), derived from benzimidazole, have demonstrated the highest level of anticancer efficacy, as shown in Table 2. Subsequently, all the derivatives underwent docking investigations against several kinase targets, including CDC-like kinases 1 to 4, epidermal growth factor receptor (EGFR) kinase, protein kinase A (PKA), and the APC-Asef interface. Figure 5 presents the findings from the structure-activity relationship (SAR) studies of the synthesized compounds. The benzimidazole ring maintains biological activity, while substitution with any equivalent bioisostere diminishes this activity. The substitution with 4,5-dimethoxy enhances the activity of the target drug, rendering it more effective than the reference compound. Substitution with halogens, such as 4,5-dichloro and 4,5-dibromo (electron-withdrawing groups), exhibits low activity. A weak electron-donating (4,5-dimethyl substitution) exhibits significant activity. A substitution at positions 4 and 5 maintains moderate activity. Substitution with 4,5-dicyano enhances anticancer activity. Electron-withdrawing groups, such as the 4,5-dinitro substituent, reduce activity. The interpretation of the results proposes that hybrid β-carbolines show promise as binders for CLK. Table 3 demonstrates the binding sites of compounds **1** and **2** to the active amino acids [68].

##### Phosphatidylinositol

Phosphatidylinositol (PI), a constituent of the cellular plasma membrane, is essential for controlling several cellular processes, such as differentiation, proliferation, phagocytosis, and chemotaxis of the cell [69]. The PI3K pathway is commonly active in human malignancies [70]. In the realm of anticancer research, there is ongoing development of inhibitors that specifically target crucial components within this pathway [71].

Previous investigations have successfully identified PI3K inhibitors with a benzimidazole scaffold, demonstrating a specific affinity towards PI3K types δ and γ. Considering this, a prior investigation was undertaken to examine the specific mechanism of δ and γ PI3K inhibition using four created inhibitors.

**Figure 6:**
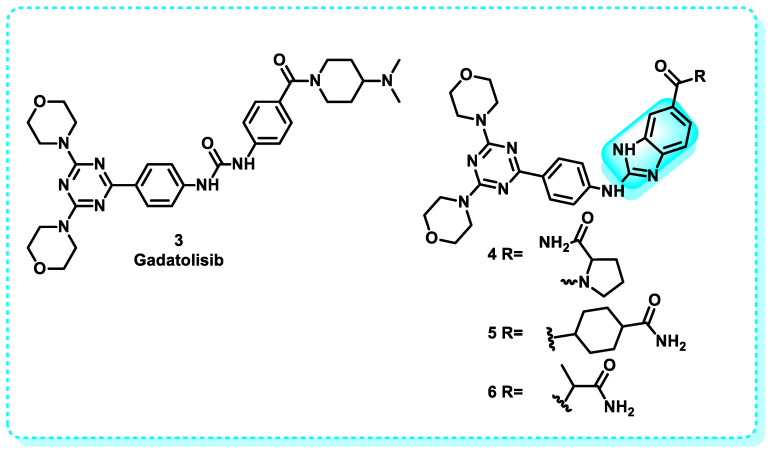
Benzimidazole derivatives act as PI3Kδ and PI3Kγ inhibitors.

This study employed various computational techniques, including key residue decomposition, molecular dynamics and docking simulation, principal component analysis, and binding free energy calculation. Gadatolisib **3** and benzimidazole derivatives **4**, **5**, and **6** were subjected to these methods, which resulted in IC**50** values of 156.0, 5.1, 2.3, and 5 µM for PI3Kδ, as well as IC_50_ values of 41.40, 444, 849, and 545 for PI3Kγ (Figure 6). The aim was to shed light on the underlying mechanisms of their behavior and highlight the binding affinity of both δ and γ PI3K. The energy decomposition analysis of the protein’s essential residues revealed that Met752, Tyr813, Asp911, Asn836, and Ile910 significantly contribute to PI3Kδ selectivity. On the other hand, MET804 and ALA805 have been discovered to be influential in determining PI3Kγ selectivity. The purpose of this research is to establish a solid understanding of the molecular foundation and dynamic characteristics that will inform the development of particular inhibitors aimed at PI3Kγ and PI3Kδ [72].

##### Checkpoint Kinase

It belongs to the family of serine/threonine kinases that govern the course of the cell cycle [73]. Checkpoint kinase (Chk1) is considered an essential regulator for maintaining cell life and progression pathways. It is necessary to control DNA damage checkpoints [74]. Normal homeostasis requires the optimal functioning of DNA checkpoints; however, recent investigations have demonstrated the overexpression of this gene in cancer cells [75]. Chk1 in cancer was shown to govern cell invasion, survival, and proliferation. Consequently, many Chk1 inhibitors are now in development to specifically target cancer cells [76].

**Figure 7:**
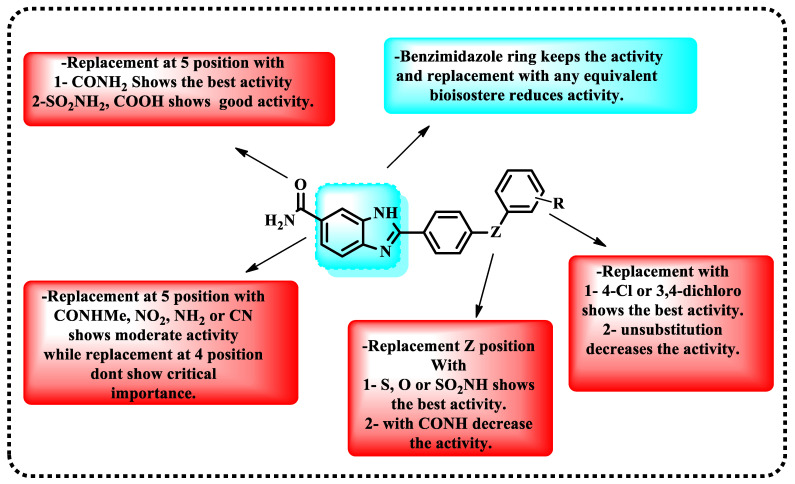
Demonstration of the structure-activity relationship of 2-arylbenzimidazole.

In this paper, a novel series of kinase inhibitors belonging to the 2-arylbenzimidazole class was reported. The first SAR of checkpoint kinases related to benzimidazole was demonstrated in Figure 7. On the basis that 5-unsubstituted benzimidazole exhibited no activity at Chk2, several analogs of this compound were examined. Minimum inhibition concentrations (IC_50_) were recorded, and the compounds were arranged in an ascending manner according to IC_50_ (Table 4). Analogs led to the discovery of a far more powerful molecule [77].

##### AMP Kinase

Adenosine monophosphate (AMP)-activated heterotrimeric protein kinase (AMPK), or a molecular energy sensor, consists of three distinct subunits: the α, β, and γ subunits, with the catalytic α and regulatory ones, β and γ, which govern the regulation of cellular metabolism [78]. When a deficiency in food levels triggers AMP-activated protein kinase (AMPK), it promotes glucose absorption and lipid oxidation to generate energy [79]. Simultaneously, it suppresses energy-consuming activities, such as glucose and fat formation, to restore energy balance. AMPK plays a crucial role in managing glucose balance throughout the body by modulating metabolic processes in several peripheral tissues [80]. AMPK mediates intertissue signaling, reacting to hormonal cues such as leptin and adiponectin. It plays a vital role in maintaining the overall energy equilibrium of the organism by coordinating communication between peripheral tissues and the hypothalamus [81,82,83]. In addition, it has been demonstrated that AMP-activated protein kinase (AMPK) has a suppressive effect on the mechanistic target of rapamycin (mTOR) and cyclin-dependent kinase (CDK) pathways and activates p53. Additionally, AMPK has been shown to trigger autophagy [84]. Therefore, the design and development of AMPK inducers can function as a practical therapeutic approach for combating different forms of cancer [82,85].

**Figure 8:**
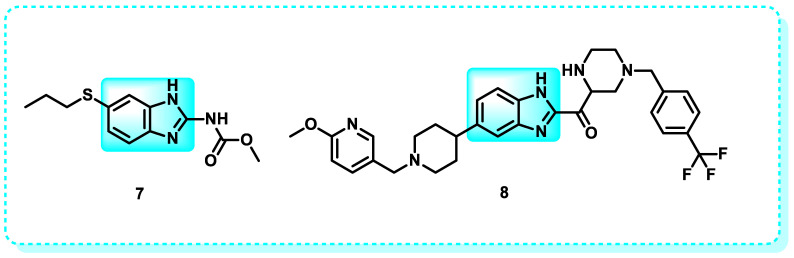
Benzimidazole derivatives act as an AMPK activator.

Examine the effectiveness of benzimidazole derivatives in tumor treatment. Specifically, the focus was on evaluating the impact of administering albendazole (ABZ) **7** on the inhibition of growth and movement of MDA-MB-231 cells as well as its capacity to trigger programmed cell death. Furthermore, the Western blotting analysis demonstrated that ABZ triggered programmed cell death in MDA-MB-231 cells via the GLUT1/AMPK/P53 signaling cascade [86]. Further optimization endeavors resulted in the discovery of compound **8**, named ASP4132, a benzimidazole derivative that exhibits high potency, moderate aqueous solubility, and oral bioavailability as an activator of AMPK. This drug shows promise as a potential precision medicine for treating several types of human malignancies (Figure 8) [73].

##### Cyclin-Dependent Kinase

These protein kinases contribute to the advancement of the cell cycle by adding phosphate groups to specific amino acid sequences in protein substrates, using ATP as a source of phosphate [87]. When these kinases malfunction, it can result in a range of diseases, including malignancy. The excessive and abnormal expression of cyclin-dependent kinases (CDKs), particularly cyclin D, is responsible for the start and advancement of tumors [88]. CDK inhibitors are presently under investigation as a possible target for cancer treatment [89].

**Figure 9:**
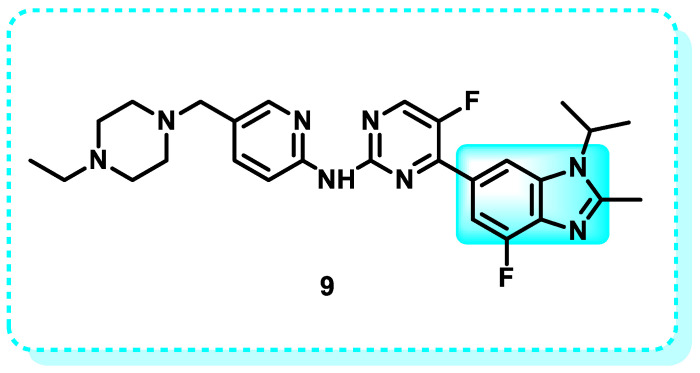
Shows that derivatives of benzimidazoles inhibit CDK.

In 2017, abemaciclib (**9**) was developed by Eli Lilly and Company. Abemaciclib is an FDA-approved cyclin-dependent kinase (CDK) inhibitor, specifically for CDK-4 and CDK-6 (Figure 9). It is employed in conjunction with endocrine therapy and serves as an adjuvant treatment for hormone receptor-positive/HER2-negative, advanced, and metastatic breast cancer [90,91]. Abemaciclib specifically targets CDK4 (with an IC_50_ of 2 nmol/L) and CDK6 (with an IC_50_ of 10 nmol/L) by competitively binding to the ATP-binding site of these enzymes. CDK4/6 inhibition impeded Rb phosphorylation, leading to cell cycle arrest in the G1 phase and inhibiting cancer cell proliferation [92].

##### Aurora Kinase

The primary members of the aurora kinase family are aurora kinases A, B, and C, which play crucial roles in several key mitotic events. However, when the activity of Aurora kinase is disrupted, it might result in aberrant mitotic activities [93]. The presence of an excessive amount of Aurora kinase has been detected in solid tumors, including lung, colon, and breast cancer, and other types of cancer [94]. Therefore, inhibiting this kinase shows potential as a therapeutic strategy in creating anticancer medication [95].

**Figure 10:**
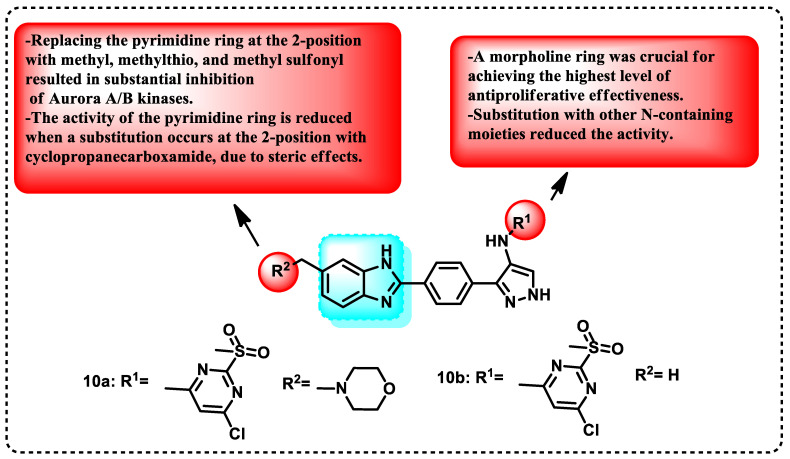
Demonstration of the SAR of benzimidazole compounds as Aurora kinase inhibitors, and benzimidazole derivatives function as inhibitors of Aurora kinases.

Many recent reviews provide an overview of the tactics employed by different researchers in designing and developing Aurora kinase inhibitors of diverse structural classes. This article reports the development, synthesis, and assessment of pyrazole-benzimidazole derivatives for their biological activity in inhibiting Aurora A/B kinases. The compounds were screened using an Aurora kinase inhibitory assay and a set of cancer cell lines (U937, K563, A549, LoVo, and HT29). Several synthesized substances exhibited strong inhibitory effects, with low levels of inhibition observed in the single-digit micromolar range against various cell lines. Compound **10a** exhibited the most potent suppression of both A and B AURK, with IC_50_ values of 28.9 and 2.2 nM for AURKA/B, respectively. Compounds **10a** and **10b** exhibited equal inhibition, as they shared the same pyrimidine substitution. The primary distinction was the inclusion of a morpholine ring in **10a**. Docking studies were conducted to justify the in vitro findings. These studies revealed that compound **10a** effectively binds to AURKA and AURKB at the ATP binding site (PDB ID for AURKA: 2W1G and PDB ID for AURKB: 2VGO). In Figure 10, the SAR related to benzimidazole compounds as inhibitors of Aurora kinase is shown. Additionally, structural analysis indicated that the benzimidazole and pyrazole configurations had the lowest energy, which supported the biological activity reported [96].

#### 2.1.2. Tyrosine Kinase

Protein tyrosine kinases (PTKs) are an extensive set of enzymes that control numerous physiological functions [97]. The primary factor enabling their extensive participation in signaling is their adjustable substrate specificity and regulatory mechanisms, which allow the enzyme to respond to unique regulatory signals and phosphorylate the appropriate physiological protein substrates [98]. Therefore, in addition to the overall PTK catalytic platform, each protein tyrosine kinase (PTK) has distinct structural patterns that combine various catalytic and regulatory attributes [97]. Activating several protein kinases in cancer cells facilitates tumor growth and progression. Protein kinases facilitate the addition of phosphate groups to the tyrosine and serine/threonine sites [99]. TKs consist of receptor tyrosine kinases (RTKs), such as EGFR, FGFR, and PDGFR, as well as non-receptor tyrosine kinases (NRTKs), including ABL, FAK, and SRC [10,100,101].

**Figure 11:**
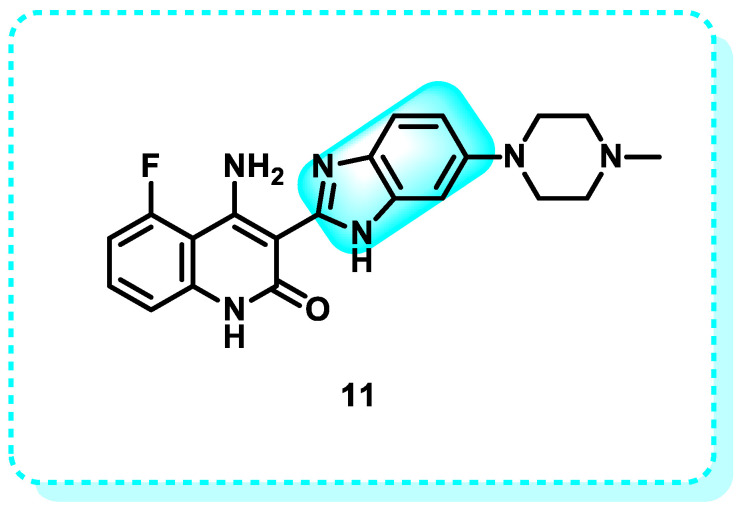
Dovitinib is an example of benzimidazole derivatives that target tyrosine kinases.

Dovitinib **11** is a chemical compound that is a highly effective pan-tyrosine kinase inhibitor that can be taken orally. It targets explicitly vascular endothelial growth factor receptor (VEGFR), fibroblast growth factor receptor (FGFR), and other tyrosine kinases (Figure 11) [102,103].

##### HeR2

The HER2/neu proteins, also known as human epidermal growth factor receptor (HER2) receptors, play a crucial role in regulating the proliferation and repair of normal breast cells [104]. In approximately 10–20% of cancer cases, a malfunction in this gene occurs, causing the gene to produce multiple copies of itself. This leads to an excessive production of the HER2 protein, resulting in unregulated cell proliferation and division [105]. Therefore, controlling HER2 proteins is regarded as a highly promising target for cancer treatment [106].

**Figure 12:**
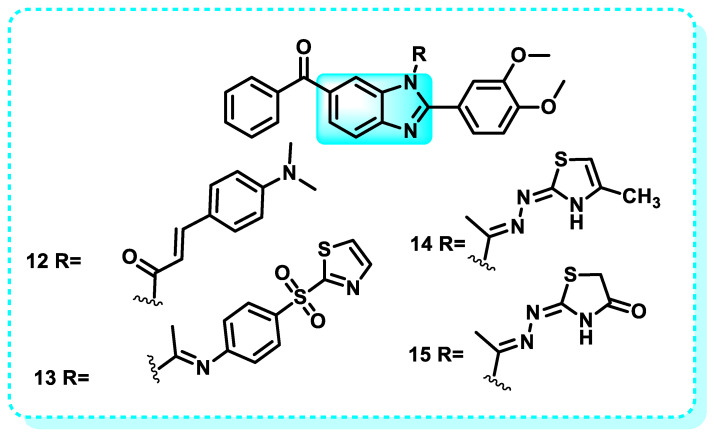
Benzimidazole derivatives that have a suppression effect on HER2.

This work focuses on the synthesis and evaluation of benzimidazole derivatives as possible anticancer drugs targeting cervical HeLa carcinoma cells. The most promising cytotoxic compounds **12**–**15**, were evaluated for their inhibitory effect against EGFR/HER2 kinases (Figure 12). These compounds exhibited a more significant suppression effect on HER2 compared to EGFR, with IC_50_ values ranging from 0.19 to 0.31 µM. In comparison, erlotinib had an IC_50_ value of 1.23 µM. Compound **13**, which is a derivative of 2,5-dioxopyrrolidine, and compound **15**, which is a thiazolidin-4-one, proved to be highly promising as dual EGFR and HER2 inhibitors [107].

##### Estrogen Receptors

The nuclear estrogen receptor (ER) regulates the function of estrogen complexes and can modulate gene expression through its interactions with other receptors and proteins [108]. To comprehend the function of estrogen receptors in both normal bodily processes and disease development, as well as to elucidate the pharmacological properties of antiestrogens, estrogens, and selective estrogen receptor modulators (SERMs), it is necessary to acknowledge the presence of two separate estrogen receptor types, namely members of the extensive nuclear receptor family, serving as hormone-dependent transcription factors that play crucial functions in the endocrine signaling system [109]. Estrogen signaling relies on many pathways involving estrogen receptors and other proteins that are not directly related to estrogen [110].

Estrogen signaling regulates the equilibrium between the ERα and ERβ receptors and their alternative splicing variants. As a result of their positive ER hormone status, breast cancer cells that have estrogen receptors (ER) use estrogen for cell growth. Treating cancer cells with anti-estrogen hormone treatment or ER inhibitors can impede their proliferation [111].

In this study, it is proposed that the growth of tumors in situations where hormone treatment is ineffective still depends on the interaction between estrogen receptor alpha (ERα) and increased levels of coactivators. Simultaneously targeting both the primary ligand binding site (LBS) and the coactivator binding site (CABS) at ERα has the potential to be a highly effective therapeutic strategy for overcoming resistance caused by mutations in breast cancer. A series of benzimidazole compounds (16–19) was designed and synthesized, exhibiting a strong binding affinity to the Erα and Erβ receptors (Table 5). Some compounds belonging to the benzimidazole series **16**–**19** demonstrated moderate RBA values, with compound **16** exhibiting the maximum affinity (RBA(ERα) = 7.52%; RBA(ERβ) = 6.78%) [93].

**Figure 13:**
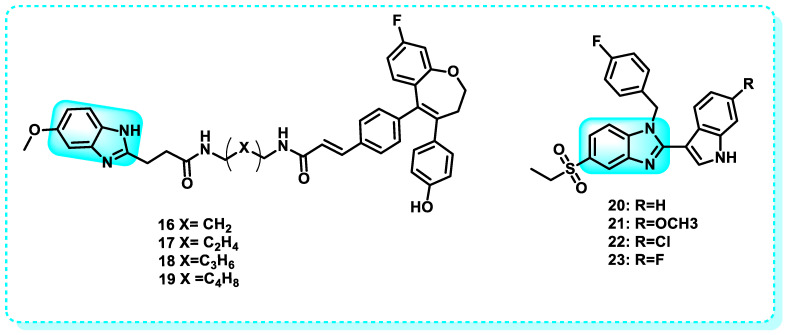
Benzimidazole derivatives that have binding affinity to estrogen receptors.

Another study indicated that the inhibitory effects on cell growth of indole benzimidazoles were more comparable between the estrogen (E2)-sensitive cell lines MCF-7 and HEPG2 than the estrogen receptor-negative (ER-) cell line MDA-MB-231. Compounds **20**–**23** were chosen as primary compounds due to their strong anticancer properties and modest structural affinity to the ER. The results indicate that these new indole-benzimidazoles can control the expression of ER target genes (Figure 13) [112].

##### EGFR Inhibitors

**Figure 14:**
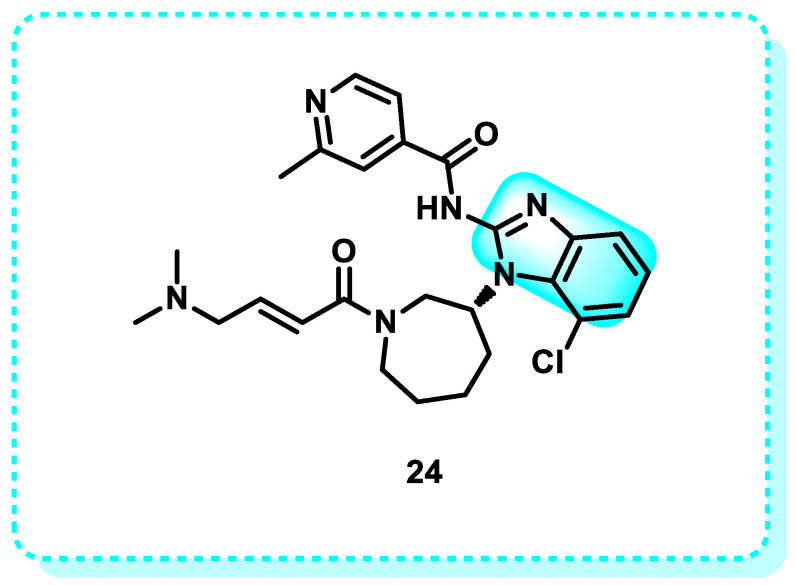
Benzimidazole derivatives (Nazartinib) as EGFR inhibitors.

The epidermal growth factor receptor (EGFR) plays a crucial role in the development of cancer. Angiogenesis plays a crucial role in the development of cancer, promoting the growth of new blood vessels and facilitating the spread of cancer cells to other parts of the body. Epidermal growth factor (EGF) and vascular endothelial growth factor (VEGF) are proven crucial components that regulate the proliferation and dissemination of tumor cells [113]. When EGF, also known as transforming growth factor (TGF), binds to the EGFR, it leads to the formation of dimers and triggers a series of subsequent signaling cascades [114]. The MAPK signaling pathway transmits crucial signals from the cytoplasm to the nucleus. It significantly regulates the cell cycle by controlling cyclin D and other cellular components involved in the genesis, advancement, endurance, growth, and spread of cancer [115]. Similarly to EGF, the tumor cell secretes an excess of VGEF, which interacts with the V and subsequently stimulates VGEFR. Subsequently, this connection triggers the process of angiogenesis, aiding in the survival, movement, and spread of tumors. Consequently, the investigation of the pathogenic function of EGFR and VEGFR has led to the development of inhibitors targeting them, which have demonstrated notable anti-tumor benefits [116]. Nazartinib (EGF816) is a benzimidazole derivative **24**, which is a small oral molecule that belongs to the third generation of epidermal growth factor receptor (EGFR) tyrosine kinase inhibitors (TKIs) (Figure 14). It is now in Phase II clinical studies, and Novartis is the developer. Nazartinib exhibited therapeutic effectiveness in individuals diagnosed with non-small cell lung cancer with the EGFR-Thr790Met mutation, including those with brain metastases at initial evaluation, as evidenced by a Phase I trial [117].

##### VEGFR Inhibitors

The vascular endothelial growth factor (VEGF)/vascular endothelial growth factor receptor (VEGFR) axis has been recognized as a key factor in tumor vascularization, playing a pivotal role in angiogenesis [118]. The FGF-FGFR signaling pathway is essential in the development and progression of cancer, and inhibitors targeting FGF/FGFR show enormous potential as a treatment for tumors with FGFR alterations. The VEGF-VEGFR signaling route is the primary mechanism for stimulating angiogenesis, and blocking this cascade has effectively treated tumors [119]. As a result, multiple approaches have been developed to target VEGF and its associated receptors, specifically for the treatment of cancer. However, medications aimed at full-length VEGF have led to a slight improvement in both overall survival and progression-free survival in different types of malignancies. Furthermore, the suppression of VEGFRs is linked to undesired off-target consequences [120].

In addition, recent studies have revealed VEGF splice variants that regulate sprouting and non-sprouting angiogenesis. Cues in the tumor microenvironment determine the expression patterns of these variations. It is essential to note that these variations’ modes of action contradict the established norm of VEGF signaling [121].

ABZ (**7**) suppresses the release of VEGF in ovarian cancer cells [122]. Furthermore, the utilization of ABZ in conjunction with radiation led to a synergistic enhancement in the susceptibility of lung (H153 and H446) and skin (A375 and A2058) cancer cells to radiation [123,124]. ABZ augmented radiation-induced apoptosis by a dual mechanism involving cell cycle arrest in the G2/M phase and the development of DNA damage. In contrast, paclitaxel only enhances the sensitivity of melanoma to radiation by inducing a halt in the progression of the cell cycle, specifically during the G2/M phase [123].

##### C-MET-Kinase

**Figure 15:**
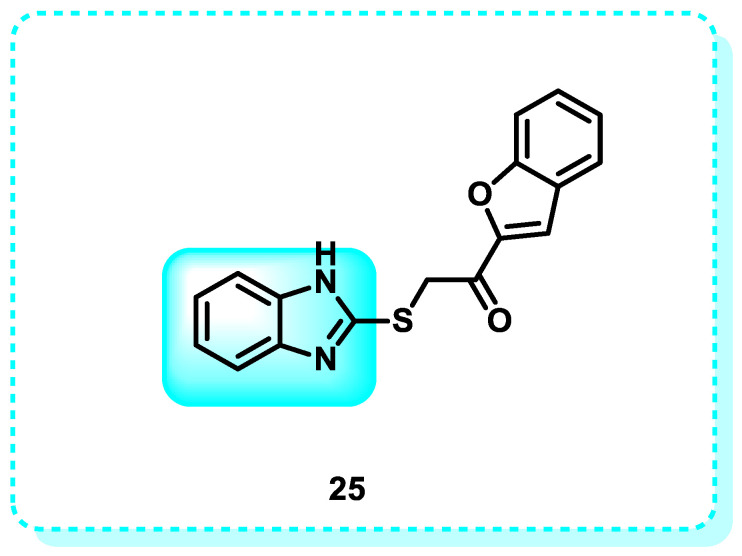
Benzimidazole derivatives that target C-MET-Kinase.

The term “C-mesenchymal–epithelial transition receptor” refers to the protein receptor known as c-Met. Stimulating the c-Met kinase, a receptor tyrosine kinase encoded by the proto-oncogene MET, leads to the movement, growth, and formation of new blood vessels in cells. However, when the MET pathway is deregulated, it can result in many disorders, including cancer. Therefore, c-Met emerges as a promising candidate for cancer therapy [125]. The c-Met gene is in the chromosomal region 7q21-31 and comprises 24 exons. However, the promoter region is situated on chromosome 1. Epithelial cells express c-Met. The c-Met receptor is a transmembrane tyrosine kinase receptor composed of two subunits, alpha and beta, connected by disulfide bonds [126]. In this study, the benzimidazole series was produced and evaluated for its anticancer efficacy against the A498 kidney carcinoma cell line. Out of all the compounds examined, compound **25** had the most favorable IC_50_ value of 6.97 µM, which is slightly better than that of the reference medication sunitinib, with an IC_50_ value of 6.99 µM (Figure 15). Additionally, an investigation of the cell cycle revealed that compound **25** resulted in a significant decrease in the number of cells in the G2/M phase. Furthermore, it was found to have a late apoptotic induction effect, as demonstrated by the Annexin-V FITC study. The enzymatic inhibitory analysis of compound **25** against c-Met and MAP kinases demonstrated its exceptional activity against c-Met kinase, with an IC_50_ value of 0.27 µM. This activity was higher than sunitinib, which had an IC_50_ value of 0.18 µM. Ultimately, the results were corroborated by a molecular docking analysis of compound **25**, which revealed its potential contacts at the active site of the c-Met kinase enzyme [127].

### 2.2. Topoisomerase

DNA topoisomerases are vital enzymes present in living creatures that have a crucial function in DNA metabolism and keeping the genetic material in its optimal condition [128]. They regulate, preserve, and alter DNA structure during replication, transcription, translation, recombination, and chromosome separation [129,130]. Two categories of DNA topoisomerases exist: Type I topoisomerase (Topo I) specifically breaks one strand of the DNA molecule. Conversely, type II topoisomerase (Topo II) operates by cleaving both strands of the double-stranded DNA molecule [130].

In this study, a series of compounds derived from benzimidazole and oxadiazole were produced and evaluated for their anticancer properties against specific lines of cancer cells: HeLa, MCF7, A549, HepG2, and C6. Compounds **26** and **27** had superior antiproliferative activity compared to Hoechst 33342 and doxorubicin against the HeLa cell line, with IC_50_ values of 0.224 ± 0.011 µM and 0.205 ± 0.010 µM, respectively. Moreover, the efficacy of these decisive lead cytotoxic agents was assessed regarding their inhibitory effectiveness against topoisomerase I, and it was shown that the chosen molecules can inhibit topoisomerase I effectively [131].

**Figure 16:**
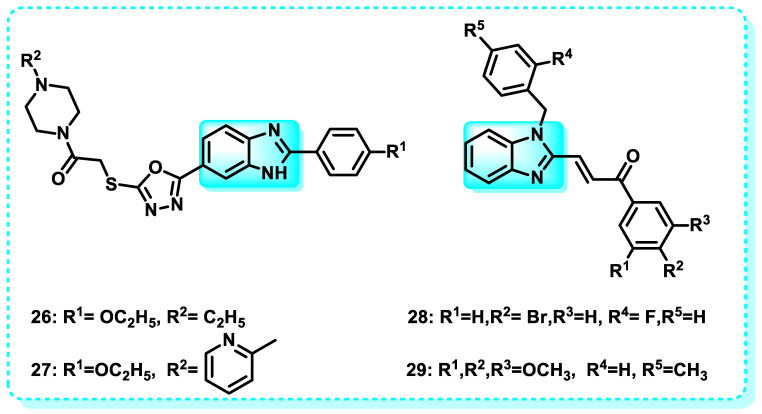
Benzimidazole derivatives as DNA topoisomerases.

Another study reveals that benzimidazole-chalcone hybrids (BCHs) are bioactive chemical compounds that exhibited significant inhibitory activity in the DNA relaxation experiment mediated by topoisomerase II and demonstrated anti-proliferative effects in four tumor cell lines. Among the compounds tested, **28** and **29** exhibited the highest potency, with IC_50_ values below 5 μM, surpassing etoposide. Mechanistic studies revealed that the BCHs acted as non-interactive catalytic inhibitors of Topo II. In addition, both **28** and **29** exhibited multifaceted characteristics in combating tumors. This includes their ability to hinder the development of colonies and the migration of cells, as well as their capacity to enhance the process of apoptosis in A549 cells (Figure 16) [124].

### 2.3. Tubulin Polymerization

Polymers (α and β-tubulin subunits) make up the primary cytoskeleton component known as microtubules (MTs), which are critical for different cellular processes, like cell motility, signaling, movement, mitotic proliferation, polarity, cell division, intracellular transport, and more [132]. The most effective targets for chemotherapy in treating cancer currently are microtubules (MTs) and their subunits α- and β-tubulin, which antimitotic medicines can inhibit [133,134]. Antimitotic medicines exert their effects by inhibiting the development of the microtubule spindle [135,136].

**Figure 17:**
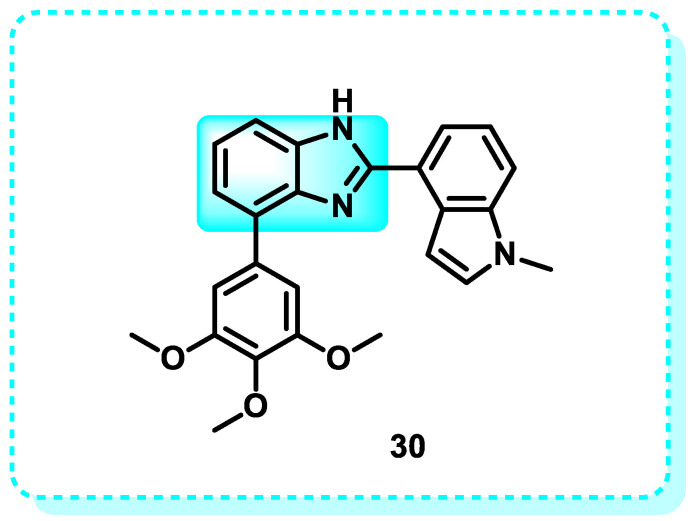
An inhibitor of tubulin polymerization, benzimidazole derivatives.

This study produced tubulin inhibitors with strong antiproliferative effects by designing and synthesizing indazole and benzimidazole analogs. Compound **30** demonstrated the most potent inhibitory effects on cancer cell development, with an average IC_50_ value of 50 µM, slightly above that of colchicine (Figure 17). Compound **30** demonstrated comparable efficacy against both a paclitaxel-resistant cancer cell line (A2780/T/T/T, IC_50_ = 9.7 µM) and the equivalent parental cell line (A2780S, IC_50_ = 6.2 µM), effectively surmounting paclitaxel resistance in vitro conditions. The X-ray crystallography technique successfully determined the crystal structure of **30** in association with tubulin at a resolution of 2.45 Å. Furthermore, the direct binding of **30** to the colchicine site was confirmed [137].

### 2.4. Telomerase Inhibitors

Telomeres are repetitive DNA sequences, known as terminal transferase, located at the ends of linear chromosomes [138]. Telomeres are protective sequences that stop DNA from being cut or merged with neighboring chromosomes [139]. Every time a cell divides, the telomere shortens by 50–200 base pairs because the DNA strand that is now in the process of replication cannot duplicate the 3′ end of the chromosome [140,141]. However, throughout the process of carcinogenesis, the excessive activation of telomerase plays a role in enhancing the survival and multiplication of tumor cells. Moreover, many oncogenic signaling pathways, including NF-kB, c-MYC, PI3K, Akt, and Wnt, have been found to induce telomerase activation [142].

Anticancer treatment strategies that target telomerase have thus far shown promise. Various small compounds have been developed with the aim of inhibiting telomerase activity, which could lead to the eradication of cancer cells while minimizing any harmful effects on healthy cells [143].

This study reveals that benzimidazole derivatives exert a suppressive effect on telomerase activity and promote the stability of the G-quadruplex structure. Compound **31** exhibited superior activity compared to the other produced compounds, which is attributed to the inclusion of a linker. Adenine and guanine were able to make hydrogen bonds with the benzimidazole ring’s -NH and -OCH_3_ groups with the help of this linker. The chemical exhibited a substantial 50% suppression of DNA synthesis within the 1–3 μM concentration range [144].

**Figure 18:**
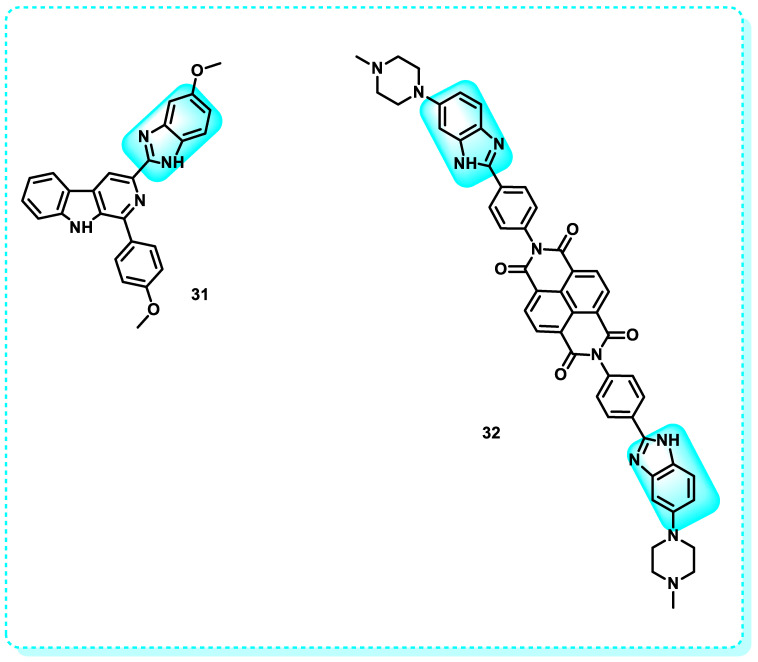
Benzimidazole derivatives act as tubulin telomerase inhibitors.

Further work demonstrated the production of bis-benzimidazole naphthalene di-imide ligands that successfully stabilized the telomeric region of human G-quadruplex DNA. Research findings showed that the administration of compound **32** effectively suppressed telomerase activity (with an IC_50_ value of 4.56 μM) and triggered programmed cell death (apoptosis) in cancer cell lines while causing minimal harm to normal HEK293T cells (Figure 18). The candidate demonstrated significant anticancer activity against A549 (IC_50_ = 19.7 μM), HeLa (IC_50_ = 20.5 μM), and HEK293T (IC_50_ = 63.4 μM) cells, making it the most powerful contender [145].

### 2.5. Apoptotic Proteins

**Figure 19:**
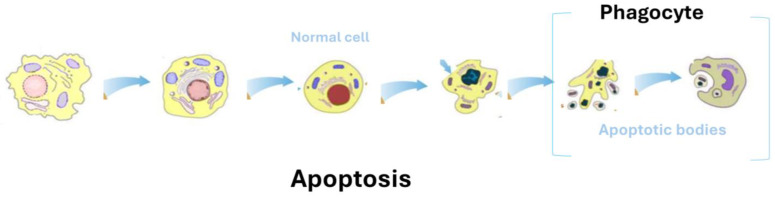
An illustration of the necrosis and apoptosis processes.

Apoptosis can be distinguished from alternative modes of cell demise, such as necrosis, which can be triggered by several factors, including toxins, physical stimulation, or ischemia [146]. Prominent characteristics of necrosis include cellular swelling and membrane breakdown, accompanied by the lysis of nuclear chromatin rather than its condensation [147]. In contrast, the occurrence of membrane damage in apoptosis is typically delayed, and the subsequent engulfment of dead cells by adjacent cells or phagocytes results in minimal or negligible inflammation, as seen in Figure 19 [131]. The presence of characteristic apoptotic morphology can be observed in several scenarios, such as the elimination of cells during the process of development, the homeostatic management of cell populations, and the aging process (Figure 19) [148,149,150]. Somatic cells undergo mitosis for production and apoptosis for termination. Maintaining a precise equilibrium between the processes of mitosis and apoptosis is crucial for the health of all bodily systems. However, disruption of the equilibrium, specifically a decrease in the rate of apoptosis, triggers the onset and progression of cancer [135].

**Figure 20:**
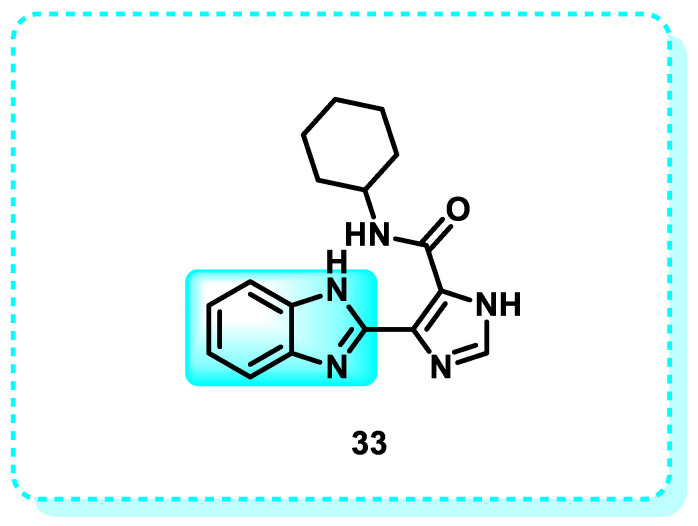
Benzimidazole derivatives act as inhibitors of autophagic flux.

According to this research, one method of cancer treatment is the stimulation of apoptosis by activating several genes and proteins while simultaneously suppressing anti-apoptotic genes and proteins. Compound **33** is an effective inhibitor of autophagic flux. It has demonstrated potent activity against human triple-negative breast cancer (TNBC) cell lines MDA-MB-231 and MDA-MB-468, with IC_50_ values of 8.3 μM and 6.0 μM, respectively (Figure 20). These values are comparable to the potency of 5-FU and cisplatin, the primary treatments for TNBC. Consistently, the application of compound **33** to TNBC cells resulted in a reduction in the levels of the anti-apoptotic protein Bcl-2. At the same time, the expression of pro-apoptotic proteins, including Bak, Bim, and Bax, increased in a manner that depended on the dosage. Furthermore, administering compound **33** to both MDA-MB-231 and MDA-MB-468 cells increased the levels of cytochrome c, cleaved caspase-3, cleaved caspase-9, and cleaved PARP in a dosage-dependent manner [136].

#### 2.5.1. p53

P53, sometimes called tumor protein (TP53), is a genome guardian [151]. This gene plays a crucial role in regulating cell cycle progression and is an essential regulator of tumor suppressor genes [152]. When p53 is mutated or deleted, the tumor suppressor function is lost, which has been associated with tumor growth [153]. Inactivated p53 leads to uncontrolled cell growth and DNA damage. This phenomenon stimulates the proliferation of cancerous cells by causing unregulated cellular division [152,154].

**Figure 21:**
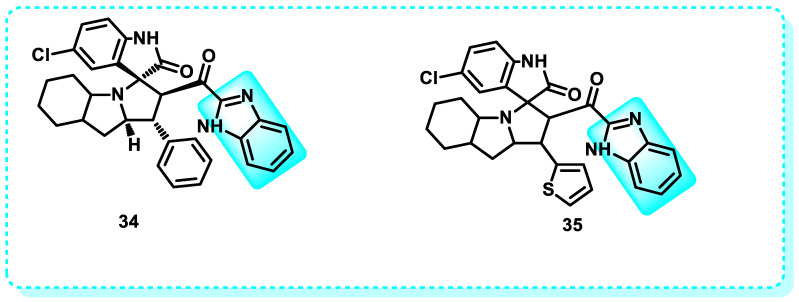
Benzimidazole derivatives that induce the p53 protein.

A novel lead compound (spirooxindole-based benzimidazole) was successfully designed and synthesized in this research and developed to be an anticancer agent. Compound **34** demonstrated therapeutic promise against different cancer cells, including the triple-negative breast cancer line (MDA-MB-231), lung cancer, prostate cancer (PC3), colon cancer (HCT-116), and A549. The safety compound **34**, as a therapeutic for normal human lung (WI-38) cells, was greater than 81% when incubated at a concentration of 5 µM. At this concentration, compound **34** caused cell death in WI-38 cells by over 50%. Compound **34**, a pharmaceutically acceptable agent, can induce the expression of genes such as p21, cyclin-D, and nuclear factor-kappa B (NF-κB) in cancer cells (Figure 21). This can be observed through the immunohistochemistry detection of the apoptotic marker p53. Its antimetastatic properties allowed it to halt the progression of breast cancer successfully [155]. The MDM2–p53 protein–protein interaction inhibitor is a highly researched subject that has garnered significant interest recently [156]. Inhibiting the link between p53 and MDM2 results in the reactivation of p53. This reactivation leads to several functions of p53, such as DNA repair, apoptosis, cell cycle arrest, senescence, metabolic changes, and tumor suppression. Compound **35** exhibited promising anticancer activity, with an IC_50_ value of 3.797 ± 0.205 µM (Figure 21). It was identified as the most active contender in the series. The investigation of MDM2 binding revealed that compound **35** effectively inhibits MDM2, with a dissociation constant (KD) of 2.38 µM. This discovery has the potential to be valuable for the advancement of cancer research in the future [157].

#### 2.5.2. BAX

BAX, a constituent of the Bcl-2 gene family, is pivotal in promoting the liberation of cytochrome C and initiating the process of apoptosis, a form of programmed cell death. BAX possesses the capability to permeabilize the outer mitochondrial membrane, thereby mediating the occurrence of apoptotic cell death [158,159,160]. The proteins BAX, BCL-XL, and BCL-2 are essential for controlling the intrinsic apoptotic process [161]. BAX is a protein of 192 amino acids with a molecular weight of 21 kilodaltons. As a tumor suppressor, it makes cancer cells more vulnerable to the effects of anticancer drugs; it is ubiquitous in healthy tissues [162,163].

**Figure 22:**
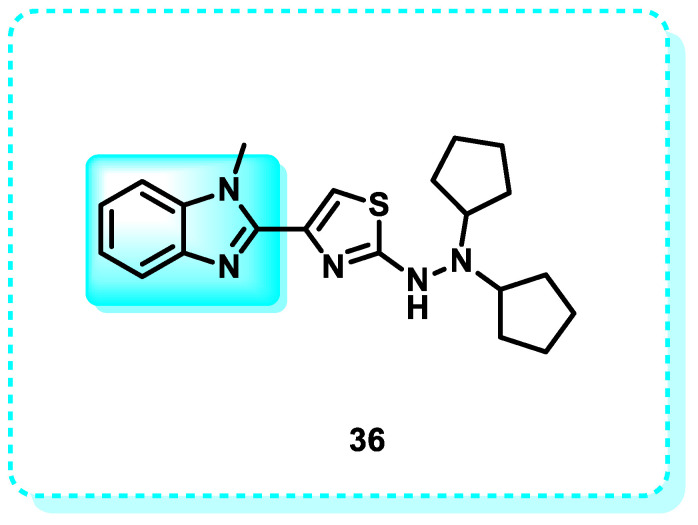
Benzimidazole derivatives act as inducers of BAX.

In this study, compound **36** elicited an apoptotic response and halted the G2/M phase of the cell cycle, thereby impeding the mitotic cycle in MCF-7 cells. In addition, **36** increased the expression of oncogenic markers, including caspase-3, p53, and Bax/Bcl-2, while also suppressing the activity of the PARP-1 enzyme (Figure 22) [164].

#### 2.5.3. Caspase-3

Caspase 3, a type of cysteine aspartyl protease, is characterized as an aspartate-specific cysteine protease. It is widely recognized as the “executioner caspase” [165]. It is essential for coordinating biological processes that lead to the disintegration of cellular structures, including the breakage of DNA and the degradation of cytoskeletal proteins [166]. Under typical circumstances, the activity of caspase-3 is essential in maintaining the balance between cellular survival and programmed cellular death (apoptosis). On the other hand, it has been noted that caspase-3 expression is declining throughout the process of carcinogenesis. This decrease in caspase-3 expression enables cancer cells to undertake continuous cell division and uncontrolled cell growth without interruption. Various apoptotic inducers, including caspase-3, have been designed and developed as anticancer drugs [167].

**Figure 23:**
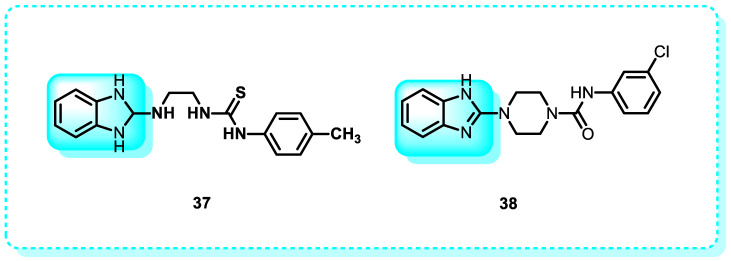
Benzimidazole derivatives act as inducers of caspase 3.

In this study, a set of urea and thiourea derivatives, incorporating a benzimidazole group, has been conceived and synthesized to develop promising anticancer medicines. Following treatment with MCF-7 cells, compounds **37** and **38** triggered apoptosis by activating caspase-3/7. Compounds **37** and **38** can trigger apoptosis in several genotypic BC cell lines. The only compounds that demonstrated cytotoxicity against the cancer cell types under investigation were **37** and **38**. The IC_50_ values for the compounds were not computed, as they exceeded 1000 µg mL^−1^. Nevertheless, compounds **37** and **38** exhibited more cytotoxicity in MCF-7 cells, with IC_50_ values of 25.8 and 48.3 µM, respectively, compared to their effects on MDAMB-231 cells. The IC_50_ values for compounds **37** and **38** on MDA-MB-231 cells were 54.3 µM and 89.5 µM, respectively (Figure 23) [161].

#### 2.5.4. Annexin V

The Annexin V superfamily comprises 13 proteins with calcium- or phospholipid-binding capabilities. These proteins share significant pharmacological and structural similarities, ranging from 40% to 60% [168]. This protein is a member of the Annexin group and is found within cells. It contributes to diverse biological functions, including cell death, cell growth, inflammation, blood clotting, blood vessel formation, cell communication, tumor development, and drug resistance. Several methods have found these proteins to be vital in tumor differentiation and development [169]. One protein that binds particularly to phospholipids is annexin V, and it is calcium-dependent. It controls the activity of protein kinase C (PKC). Annexin V suppressed the phosphorylation of annexin II by naturally occurring PKC and the phosphorylation of myelin basic protein by PKCα [170].

**Figure 24:**
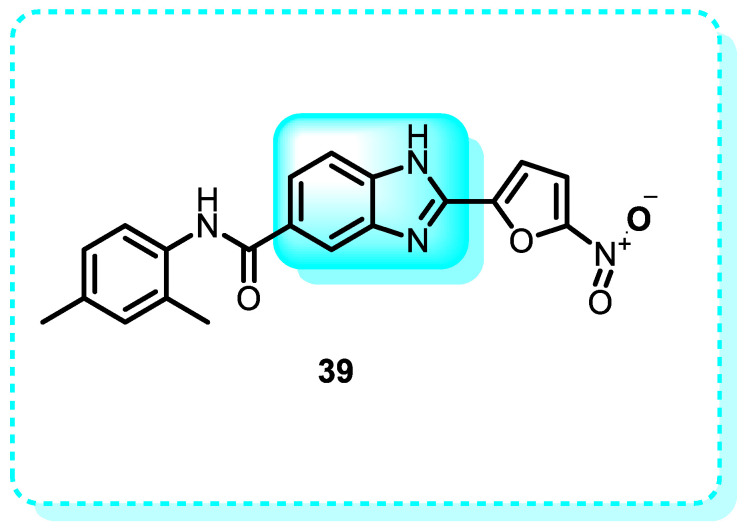
Benzimidazole derivatives that are annexin positive.

In this study, compound **39** exhibited increased toxicity against Jurkat E6.1 and HL60 cell lines while having no impact on fresh lymphocytes (Figure 24). Flow cytometry analysis revealed apoptotic cell death after 24 h. This compound effectively promoted apoptosis in both tumor cell lines by suppressing autophagy, decreasing cell survival [171].

#### 2.5.5. Apoptosis Inducers

The pro-apoptotic effects of apoptosis inducers have been discovered through various mechanisms, including DNA cross-linking, the deactivation of anti-apoptotic proteins, and the activation of caspase enzymes. The agents selectively target specific cellular pathways by providing an anticancer or antineoplastic impact [172,173].

In this study, imidazothiadiazole-benzimidazole conjugates were designed and assessed for their cytotoxicity against four specific human cancer cell lines. Compounds **40** and **41** showed noteworthy antiproliferative activity in the ME-180 (cervical) cell line. Flow cytometric research revealed that these two chemicals halted the progression of the cell cycle, specifically in the G0/G1 phase, resulting in the decline of mitochondrial membrane potential and subsequent apoptotic cell death. In addition, the staining method using Hoechst 33258, the DNA fragmentation assay, the Annexin V staining assay, and the caspase-3 analysis all indicated that compounds **40** and **41** caused cell death through apoptosis [174].

**Figure 25:**
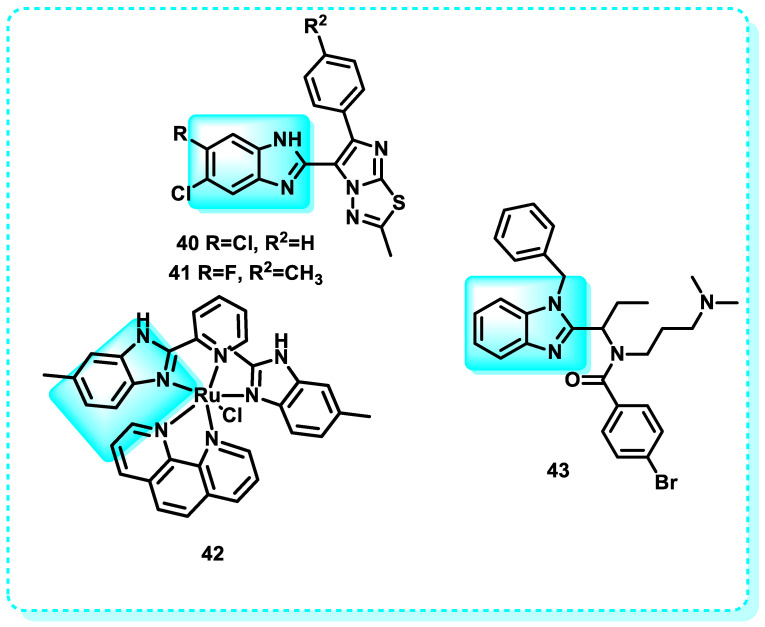
Benzimidazole derivatives, which are annexin-positive apoptosis inducers.

Another study reveals that when A375 and MCF-7 cells were exposed to varying concentrations of complexes **42** for 72 h, distinct proportions of cells underwent apoptosis. Specifically, ruthenium complex [Ru(bmbp)(phen)Cl]ClO_4_ (RuBmP; bmbp = 2,6-bis(4-methylbenzimidazol-2-yl)pyridine) **42** (RuBmP) had the highest apoptosis-inducing efficacy against cancer cells. When the A375 cells were treated with RuBmP, there was a gradual increase in the cells undergoing programmed cell death, indicated by the sub-G1 cell population (Figure 25) [175].

Additional studies indicate that the synthesis of the benzimidazole-based compound known as kinesin spindle protein **43** may serve as a new inducer of apoptosis in HCT116 cells. It has a strong inhibitory effect against kinesin spindle protein. HCT116 cells treated with CPUYJ039 were induced into mitotic arrest, characterized by the production of monogastric spindles, followed by cell death (Figure 25) [176].

#### 2.5.6. Poly ADP Ribose Kinase

PARP1 is an enzyme that facilitates the addition of a ribose group to adenosine diphosphate (ADP), a process known as ribosylation. This modification is involved in various cellular functions [177]. Poly(ADP-ribosyl)ation, alternatively referred to as PARylation, is a protein modification that can be reversed. It is performed by enzymes called poly(ADP-ribose) polymerases (PARPs), together with enzymes that break down poly(ADP-ribose) (PAR), such as PAR glycohydrolase (PARG) and ADP-ribosyl hydrolase 3 (ARH3) [178]. Poly ADP-ribose polymerase (PADP) enzyme has been the subject of intense research due to its crucial involvement in the DNA repair pathway. Several cancer types have been shown to exhibit overexpression, which facilitates the survival of cancer cells. Therefore, numerous PADP inhibitors have been investigated for their potential as a highly effective anticancer treatment [179].

**Figure 26:**
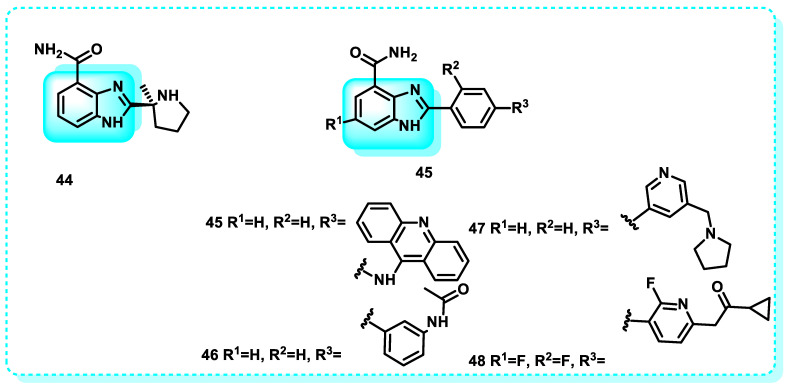
Benzimidazole derivatives, which are poly ADP ribose kinase inhibitors.

In this study, Veliparib **44**, a benzimidazole derivative, is an orally administered compound that inhibits the activity of poly (ADP-ribose) polymerase (PARP)-1 and -2. Cancer may upregulate PARP-1 expression, resulting in resistance to cytotoxic drugs. Veliparib specifically targets cell lines from breast cancer (Figure 26) [180,181].

Another study shows that compound **45** exhibits potent inhibition of PARP1 (IC50 = 0.09 µM) and establishes essential hydrogen bonds with the residues Gly863, Tyr907, and Tyr889 of PARP1. Additionally, it forms a cleavage complex with Topo and DNA. In vitro, cytotoxicity testing results demonstrated that compound **45** had more anti-proliferative activity against MCF-7 cells than Olaparib. Compound **45** had an IC_50_ value of 12.01 µM, while Olaparib had an IC_50_ value of 36.24 µM (Figure 26) [177].

An additional study was conducted in which compounds **46**–**48** were synthesized and subsequently subjected to screening. The carboxamide group is critical for the inhibitory activity of PARP1. The IC_50_ values against PARP were determined to be 0.46 µM for compound **46**, 0.061 µM for compound **47**, and 0.084 µM for compound **48** (Figure 26) [182,183].

### 2.6. Aldose Reductase

Aldose reductase 2 (ALR2) controls essential metabolic processes during normal homeostasis. However, in pathological circumstances such as diabetes, ALR2 becomes dysfunctional, resulting in secondary diabetic problems. ALR2 inhibitors are a new focus for treating diabetes-related retinopathy, specifically cataracts [184]. According to recent research, aldose reductase is an intermediary for inflammatory signals, which many stimuli, such as chemokines, growth hormones, cytokines, and carcinogens, can generate. Aldose reductase belongs to the aldo-keto reductase protein class. Persistent inflammation is a key factor in advancing cancerous growths, underscoring the criticality of focusing on aldose reductase in cancer treatment and scientific investigation [185,186].

**Figure 27:**
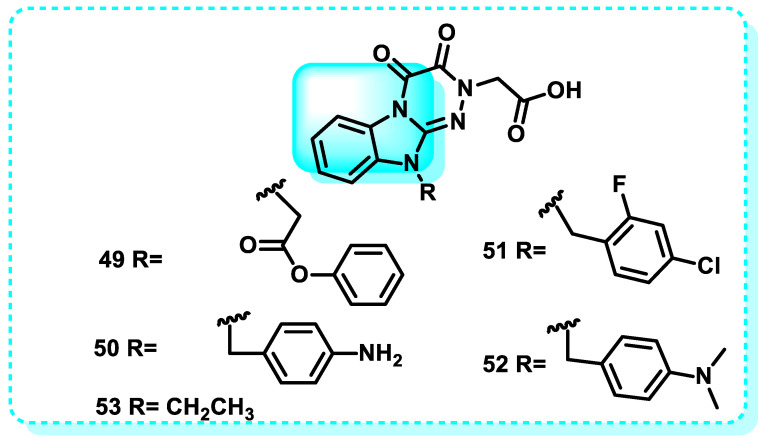
Benzimidazole derivatives are compounds that inhibit aldose reductase.

In this study, compound **49**, with an IC_50_ value of 0.32 μM, is a more potent inhibitor of ALR 2 compared to sorbinil. The phenylacetate group in Compound **49** contributes to its increased potency. However, when the R group is substituted with anilines, compound **50**, 2-fluoro 2-fluoro-4-chloro-benzyl compound **51**, or isopropyl benzylamine compound **52**, the ALR 2 activity is reduced (IC_50_ values of 32.42, 36.46, and 38.42 μM, respectively). Replacing the R position with a small alkyl group results in compound **53**, which exhibits the lowest activity level (42.40 μM) (Figure 27) [184].

### 2.7. Carbonic Anhydrase

It has been found that higher vertebrates contain a minimum of fourteen different isoforms of carbonic anhydrase (CA), which are zinc enzymes that perform vital physiological activities [187]. Several of these isozymes are located in the cytosol: CA I, CA II, CA III, and CA VII. On the other hand, CA IV, CA IX, CA XII, and CA XIV are attached to the cell membrane [188]. CAVs are found in the mitochondria, while saliva is the reservoir for CAVI. Additionally, three catalytic versions are known as CA-related proteins (CARPs): CARP VIII, CARP X, and CARP XI. Many CA isozymes play crucial roles in various physiological and physiopathological activities [189]. These enzymes are encoded by a minimum of four separate gene families [190]. It regulates the production of protons during the catalysis of the interconversion of bicarbonate and carbon dioxide. The expression of carbonic anhydrase isozyme IX (CA IX) is significantly increased in hypoxic malignancies. The inhibition of carbonic anhydrase IX (CA-IX) results in a significant reduction in the rate of growth for both primary and metastatic cancers. Hence, carbonic anhydrase inhibitors have emerged as promising candidates for the therapeutic intervention of cancer [191].

**Figure 28:**
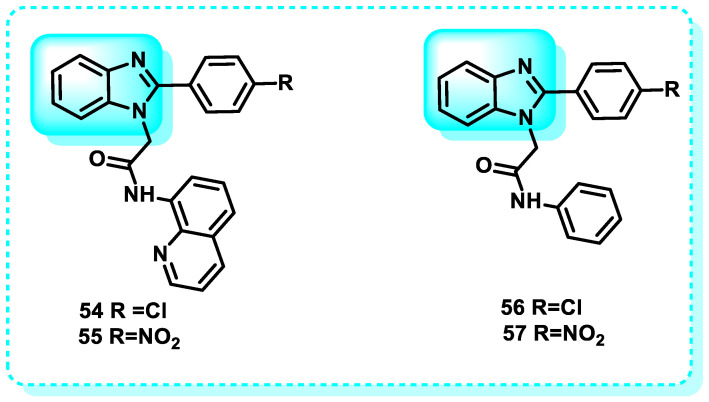
Benzimidazole derivatives, which are carbonic anhydrase inhibitors.

The purpose of this research was to examine the inhibitory effects of four benzimidazole acetamide derivatives on human erythrocyte carbonic anhydrase I (hCA-I) and II (hCA-II). The IC_50_ values for hCA-I were determined to be 7.21 μM, 4.72 μM, 6.08 μM, and 8.23 μM for compounds **54**, **55**, **56**, and **57**, respectively. In contrast, the IC50 values for hCA-II were found to be 8.64 μM, 7.07 μM, 4.12 μM, and 5.93 μM for compounds **54**, **55**, **56**, and **57**, respectively; furthermore, these values indicate that the four compounds had significant inhibitory effects on both AChE and hCA-II (Figure 28) [192].

### 2.8. COX Inhibitors

Prostaglandin production relies heavily on the COX-1 and COX-2 enzymes, which have a role in the start and course of inflammatory reactions as well as the onset of fever [193]. The COX-2 enzyme is accountable for initiating cancer stem cell-like activity and facilitating resistance to apoptosis, inflammation, angiogenesis, uncontrolled cell growth, invasion, and metastasis in cancerous cells [23].

**Figure 29:**
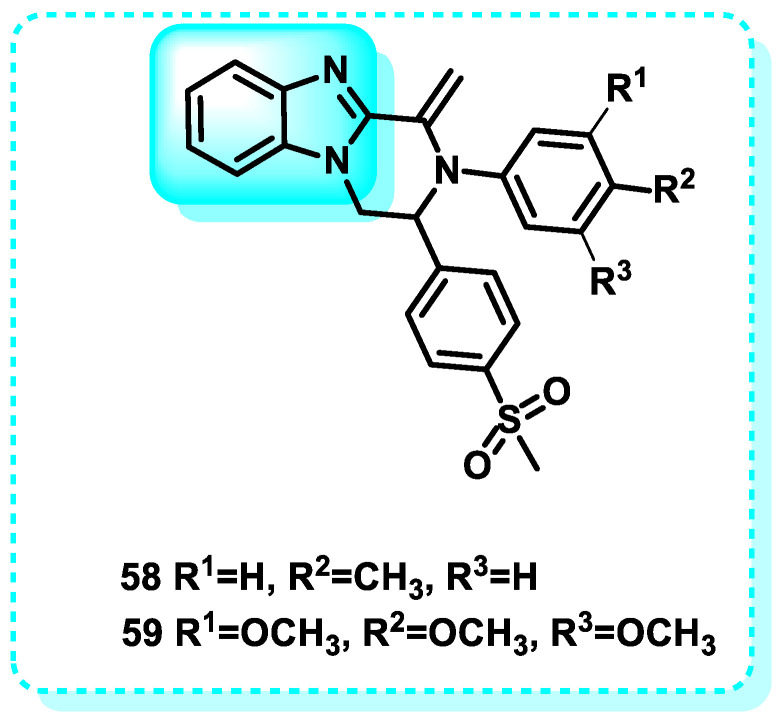
Benzimidazole derivatives, which are COX inhibitors.

In this study, a pyrazino [1,2-a]-benzimidazole set was designed and synthesized as a COX-2 inhibitor. The developed compounds exhibited selectivity as inhibitors of the COX-2 isozyme. The study also examined the effects of the substance on preventing blood platelets from clumping together and inhibiting the growth of MCF-7 cancer cells. The results indicated that the substance had satisfactory activity in both areas. The results demonstrated that compound **58** exhibited the best potency as a COX-2 inhibitor, with an IC_50_ value of 0.08 μM. Additionally, compound **59** displayed the highest selectivity index (SI = 909) (Figure 29) [194].

### 2.9. CD 133 Antigen

CD133, often referred to as prominin-1, is the predominant cell surface antigen employed for identifying and separating cancer stem cells (CSCs) in many types of solid tumors, such as those found in the brain, colon, pancreas, prostate, lung, and liver. It primarily resides in plasma membrane protrusions and regulates lipid content, cell polarity, and motility [195,196]. The significance of CSCs in the development and progression of leukemias and solid tumors is recently gaining more attention [197,198]. The occurrence of cancer stem cells (CSCs) in specific tumor types is associated with elevated expression levels of the stem cell surface marker CD133. The CD133 antigen has proven to be a valuable biomarker for identifying malignant cells that exhibit characteristics often associated with stem cells in various forms of human malignancies. Consequently, developing future treatments targeting cancer stem cells may be feasible using this marker [199,200].

**Figure 30:**
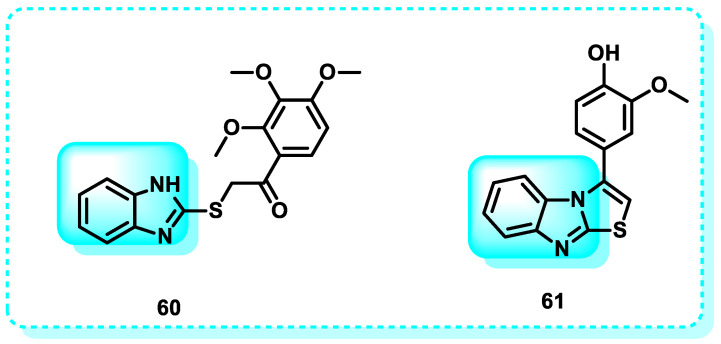
Benzimidazole derivatives, which are CD133 inhibitors.

In this study, benzimidazole derivatives were synthesized to provide a potent anti-tumor substance that could effectively attack cancer stem cells and most tumor cells. The anti-proliferative efficacy of all substances was assessed against the colon HT-29 cancer cell line. Furthermore, they can block the cell surface expression of CD133, a highly influential marker for cancer stem cells (CSCs). Compound **60** was the most effective in inhibiting cell proliferation in HT-29, with an IC_50_ value of 18.83 ± 1.37 µM. It exhibited similar potency to 5-fluorouracil (IC_50_ = 15.83 ± 1.63 µM) and had a 50.11 ± 4.05% inhibitory effect on CD133 expression (Figure 30) [201].

Another study reveals the design and synthesis of benzimidazoles to develop a very effective anticancer drug capable of targeting many tumor cells and cancer stem cells (CSCs). The synthesized compounds were assessed for their anti-proliferative activity against two specific cell lines: HT-29, a colon cancer cell line, and MDA-MB-468, a triple-negative breast cancer cell line. Furthermore, their ability to block the cell surface expression of CD133 was evaluated. Molecule Compound **61** has shown excellent potential antineoplastic activity against both tested cell lines (IC_50_ values of 9 and 12 μM, respectively) (Figure 30). Additionally, it exhibited the ability to reduce the cell surface expression of CD133 by 50%, indicating its potential to effectively control tumor growth by targeting both the central tumor mass and the proliferation of cancer stem cells (CSCs) [202].

### 2.10. Drugs Acting on DNA

Specific targeting of tumor cells can be achieved at the DNA, RNA, or protein levels. Chemotherapeutic drugs primarily interact with the DNA of tumors, while monoclonal antibodies and tiny compounds specifically target proteins [203]. The interactions between many potent anticancer medicines and DNA are critical in defining their biological impact [204]. Undoubtedly, DNA can be considered a macromolecular receptor for numerous potent anticancer drugs [205]. There are three methods by which an anticancer drug can form binding associations. The drug initially interacts with DNA-binding proteins that control transcription factors and polymerases [206]. Another approach entails employing RNA hybridization to bind with the exposed DNA, developing a triple helical nucleic acid structure [207]. This interaction hinders the process of transcribing genetic information into RNA. Thirdly, DNA intercalation and minor groove binding are examples of noncovalent interactions that may attach lower aromatic ligands to DNA molecules [208].

#### 2.10.1. Circulating Tumor DNA

Tumor cells release DNA fragments into the circulation, which is known as circulating tumor DNA (ctDNA) [209]. Individuals afflicted with cancer exhibit markedly increased quantities of cell-free tumor DNA in their circulation compared to individuals who are in a healthy condition [210,211]. At the early phases of tumor development, cells undergoing programmed cell death (apoptosis) and cell death due to injury (necrosis) release DNA from the primary tumor into the bloodstream [212]. However, it is essential to distinguish it from cfDNA, or cell-free DNA, which refers to DNA that circulates freely in the body and may not necessarily originate from a tumor [213].

**Figure 31:**
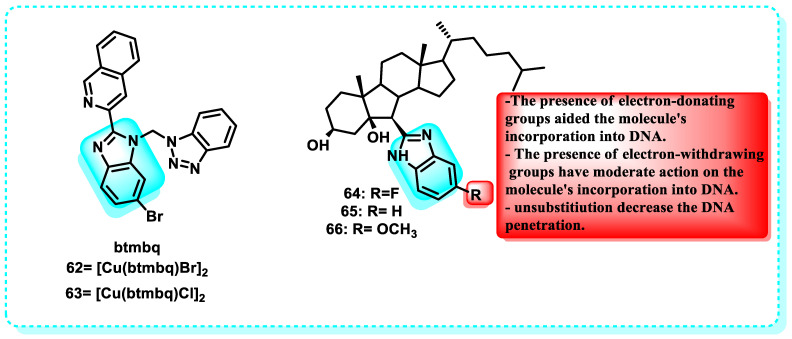
Benzimidazole derivatives which act on circulating DNA tumor and Demonstration of the structure-activity relationship of B-norcholesteryl benzimidazole.

This study examined the antitumor properties of two copper complexes, compound [Cu(btmbq)Br]_2_ (**62**) and [Cu(btmbq)Cl]_2_ (**63**), which are based on benzimidazole-quinoline. Both complexes demonstrated significant antitumor effects against the colon cancer cell line (HCT116) while exhibiting low toxicity towards the normal liver cell line (L-02) (Figure 31). CD and fluorescence spectroscopy were used to determine the interaction between the complexes and DNA, and the Ksv and Kapp values were measured. These complexes cause oxidative damage to pBR322 DNA and bind to it by intercalation, according to the results [214].

This publication presents the initial report on the binding between B-norcholesteryl benzimidazole molecules and DNA. Afterwards, ultraviolet-visible fluorescence spectroscopy, CD, and viscosity measurements were utilized to analyze the changes in ct-DNA conformation brought about by these compounds. An increase in ct-DNA content resulted in a hypochromic effect and redshift, as shown by the UV-Vis analysis. The fluorescence emission peaks of the three compounds decreased in succession as the ct-DNA concentration increased, without any alteration in their positions. This finding provided further evidence that the chemicals interact, providing additional proof that they interact with DNA, forming non-fluorescent complexes. Consequently, natural fluorescence was suppressed. Out of the compounds tested, compound **66** exhibited a greater affinity for binding to DNA compared to compounds **64** and **65**. The outcome of SAR demonstrated that electron-donating groups promoted the incorporation of the chemical into DNA. Compound **66** exhibited enhanced cytotoxicity. The study’s findings showed that the cytotoxicity pattern of the chemicals against the examined cells matched their ability to bind to ct-DNA (Figure 31) [215].

#### 2.10.2. DNA

This class of anticancer drugs is commonly referred to as antimetabolites. These substances inhibit the enzymes responsible for producing DNA’s building blocks, thereby preventing DNA from functioning and potentially leading to programmed cell death, known as apoptosis [216].

**Figure 32:**
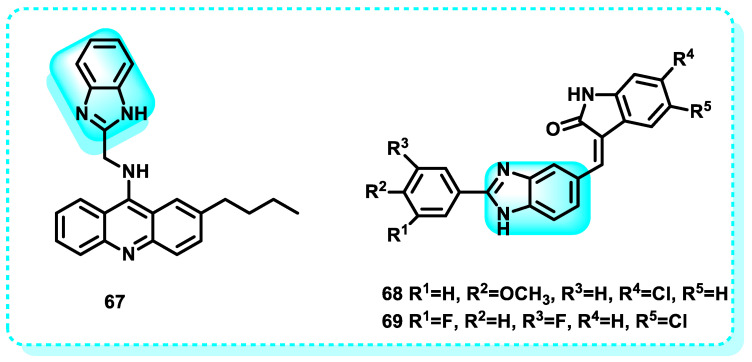
Benzimidazole derivatives that act on DNA.

A study demonstrated the synthesis of a set of benzimidazole acridine derivatives designed to create DNA-targeted compounds. These compounds were designed to intercalate in DNA and bind in the DNA minor groove, utilizing the properties of acridines and benzimidazoles, respectively. The MTT experiment revealed that most of the synthesized compounds had significant anticancer activity. Notably, compound showed the most effective results when tested against both K562 and HepG-2 cells. Subsequent investigations demonstrated that **67** had proficient DNA-binding capacity and suppressed the activity of topoisomerase I (Figure 32). Furthermore, compound **67** can trigger apoptosis in K562 cell lines via the mitochondrial pathway. The observations indicate that compound **67** has promise as a novel agent for binding to DNA and triggering apoptosis, which could be effective in treating tumors [217].

Another study showed that Compounds **68** and **69** exhibited notable antiproliferative effects on MCF-7 human breast cancer cells, indicating their specific inhibition of the uncontrolled growth of the cancer cells. In addition, apoptosis induced by **68** and **69** was identified by the depletion of mitochondrial membrane potential (DWm), production of reactive oxygen species (ROS), release of cytochrome c, fragmentation of poly ADP-ribose polymerase (PARP), triggering of caspase-9 activation, upregulation of pro-apoptotic Bax gene expression, and downregulation of anti-apoptotic Bcl-2 gene expression (Figure 32). Our findings indicate that the conjugates **68** and **69** triggered apoptosis in human breast cancer cells through the mitochondrial-mediated intrinsic apoptotic pathway [218].

#### 2.10.3. Histone Methyltransferase G9a

G9a, a histone methyltransferase responsible for facilitating the methylation process of lysine 9 on histone 3 (H3K9) and lysine 27 on histone 3 (H3K27), is increased in various forms of cancer [219,220]. Gene expression, DNA damage repair, and DNA replication are just a few of the many biological processes in which G9a plays an essential role, through its regulatory function in DNA methylation [221]. Its excessive expression has been linked to negative and unfavorable prognoses. Furthermore, it has been observed that G9a is upregulated in numerous neoplastic cells, and its presence is closely related to the initiation and progression of tumorigenesis [222]. The significant functions performed by these enzymes in different disorders have prompted the concept that these molecules serve as promising targets for future therapeutic interventions. Several G9a inhibitors show promise as cancer treatments [223].

**Figure 33:**
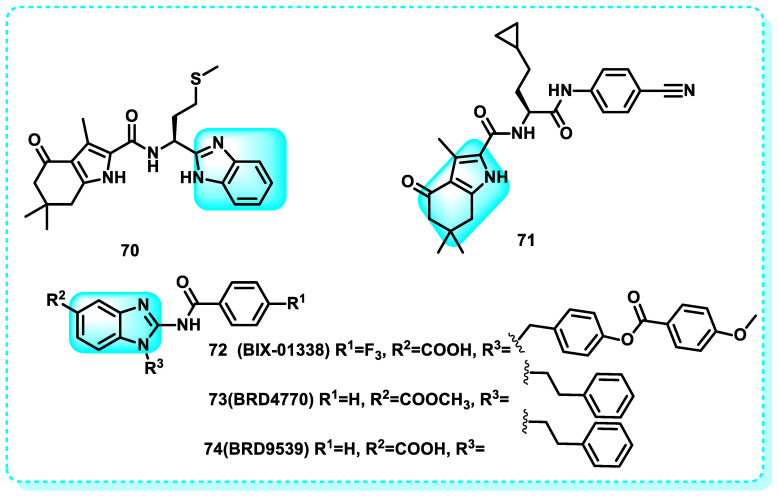
Benzimidazole derivatives act on histone methyltransferase.

This study indicated that the optimization of the in vitro characteristics of compound **70**, drug metabolism, pharmacokinetic properties, the structure–activity relationship, the help of X-ray crystallography, and fragment molecular orbital (FMO) calculations for the ligand–protein interaction resulted in the discovery of a compound called **71** (RK-701) (Figure 33). Both compounds are effective G9a inhibitors, with IC_50_ = 1.4 µM for compound **70** and IC_50_ = 0.027 µM for compound **71**. Compound **71** has shown high selectivity against other methyltransferases, a dose-dependent reduction in cellular H3K9me2 levels, and the capacity to inhibit the uncontrolled growth of cancerous cells in the MOLT-4 cell line under controlled laboratory conditions. Moreover, in a murine model of hepatocellular carcinoma (HCC) generated by carcinogens (HCC induced by carcinogens), compound **71** demonstrated the ability to inhibit tumor initiation and growth without causing any noticeable acute toxicity [224].

Another study revealed that seven successful findings were identified from a chemical library of 125,000 carefully selected compounds. Compound **72** (BIX-01338) exhibits a wide-ranging impact on all investigated enzymes, with an IC_50_ of 4.7 µM (G9a), and features a 2-(N-acyl)-aminobenzimidazole backbone, also present in other biologically active compounds. The broad-spectrum inhibitor **72** (BIX-01338) impacted methylation activity at approximately 5 µM and completely neutralized all examined enzymes at 15 µM [212,220]. Compound **73** (BRD4770) decreased the amount of di- and trimethylated H3K9 in pancreatic cancer cell line PANC-1 without causing cell death. It also triggered senescence and hindered both anchorage-dependent and -independent cell growth. The activation of the ATM pathway, which can be triggered by genetic manipulation or the use of small molecules to suppress G9a with IC_50_ = 5 µM (G9a), may be responsible for the induction of cell senescence by BRD4770. BRD4770 could serve as a valuable instrument for investigating G9a and its involvement in senescence and cancer cell biology. Furthermore, a different molecule was synthesized using the structure of BIX01338 as a basis, resulting in compound **74** (BRD9539), which has an IC_50_ value of 6.93 µM for G9a. This can elucidate the similarity in the mechanism of action between BRD4770, BRD9539, and BIX01338 (Figure 33) [220,225].

### 2.11. Oxidative Stress

When antioxidants are outnumbered by reactive oxygen species (ROS), a condition known as oxidative stress sets in. It has been associated with various diseases, including neurodegenerative diseases, cardiovascular diseases, diabetes mellitus, and other pathological conditions [226]. Oxidative stress has two facets. It is essential to maintain an appropriate level of oxidant challenge, called oxidative eustress, to regulate biological processes through redox signaling. However, an excessive quantity of oxidant challenge can cause harm to biomolecules [227,228].

#### 2.11.1. Glutathione S-Transferases

The glutathione S-transferases (GST) protein family in humans exhibits significant structural similarity and has considerable functional overlap. The enzymes can be categorized into three types based on their cellular localization: cytosolic GSTs, mitochondrial GSTs, and microsomal GSTs (also known as MAPEG GSTs) [229]. GST proteins play a vital role as antioxidant enzymes in regulating stress-induced signaling cascades. Remarkably, many hyperactive GST proteins are commonly observed in numerous human cancers [230].

**Figure 34:**
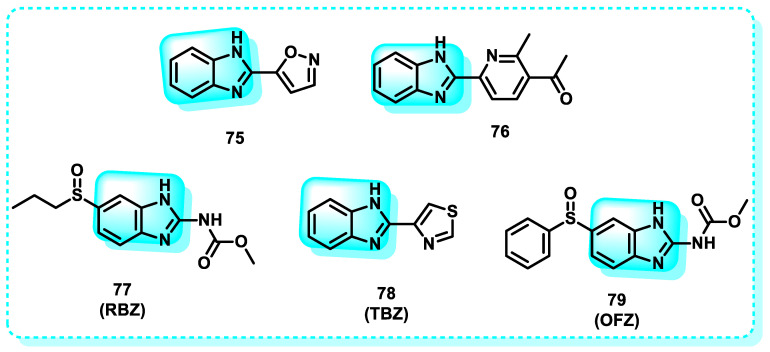
Benzimidazole derivatives act on oxidative stress.

This study presents a series of benzimidazoles fused with different heterocycles that were synthesized as inhibitors of GST (glutathione S-transferase) to serve as potential anticancer agents. The biological findings demonstrated that compounds **75** and **76** exhibited higher levels of potency as inhibitors of GST. Compounds **75** and **76** showed more potency than ethacrynic acid, with a threefold and tenfold increase, respectively. The compounds demonstrated significant cytotoxicity against breast and colon cancer cell lines. Compounds **75** and **76** exhibited strong affinity for the amino acids of the GST protein, as evidenced by molecular modeling studies. Compounds **75** and **76** met Lipinski’s rule of five, indicating their potential as novel GST inhibitors and anticancer drugs (Figure 34) [231].

Another study documented the impact of some benzimidazole medicines, including ricobendazole (RBZ) **77**, thiabendazole (TBZ) **78**, albendazole (ABZ) **7**, and oxfendazole (OFZ) **79**, on the activity of the glutathione s-transferase (GST) enzyme. The study examined the kinetics, IC_50_, and Ki values of the tested medicines on GST enzyme activity. The ordering of IC_50_ values obtained is as follows: RBZ (53.31 µM, r^2^: 0.9778) < OFZ (57.75 µM, r^2^: 0.9630) < ALBA (63.00 µM, r^2^: 0.9443) < TBZ (69.30 µM, r^2^: 0.9491). The ranking of Ki values for the GST enzyme activity of the investigated drugs (RBZ, TBZ, ALBZA, and OFZ) was 26.37 ± 2.96, 44.01 ± 5.74, 39.82 ± 3.98, and 30.14 ± 3.03 M, respectively. The experimental results demonstrated that the benzimidazole medicines examined exhibited a noteworthy inhibitory effect on the activity of GST enzymes [232].

#### 2.11.2. Thioredoxin

Members of the thioredoxin (Trx) family have essential functions in maintaining the balance of cellular redox levels [233]. Cancer cells in stressed environments depend on Trxs to safeguard themselves against the disruption of redox signaling caused by stress [234,235]. Trx-1, the most well-examined member of the family, exhibits elevated levels of numerous human malignancies, most likely as a direct result of stress [236]. Trx-1 plays a significant role in several key characteristics of cancer, such as enhanced cell division, resistance to cell death, and the accelerated formation of new blood vessels. Trx-1 is an established cancer therapeutic target linked to the proliferation of aggressive tumors, resistance to conventional treatment, and reduced patient survival [237].

**Figure 35:**
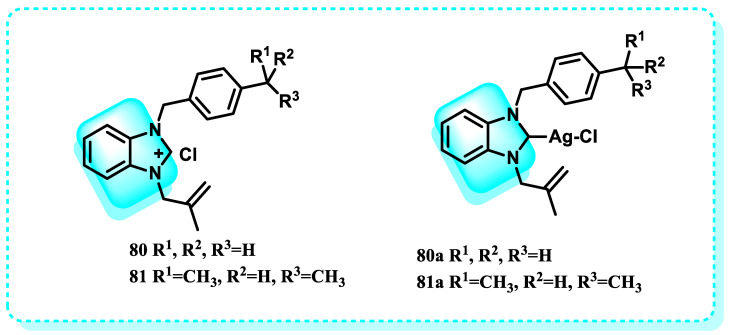
Benzimidazole derivatives that act on thioredoxin.

A study focused on examining the molecular docking of previously synthesized and characterized molecules, compounds **80** and **81**, as well as their Ag(I)-NHC complexes (**80a** and **81a**), for thioredoxin reductase (Figure 35). Furthermore, the interaction between these compounds and DNA was assessed. The binding energy of **81a** is −8.95 kcal/mol, the highest among all other compounds. It specifically interacts with the residues Ile10, Phe254, Ala38, and Val41 in thioredoxin reductase. Furthermore, the ligands exhibited interactions with Cyt11, Gua10, Cyt9, and Thy8, whereas the complexes showed interactions with Ade5, Ade6, Thy7, and Thy8, which are specific regions of the DNA [238].

#### 2.11.3. Mitochondria

**Figure 36:**
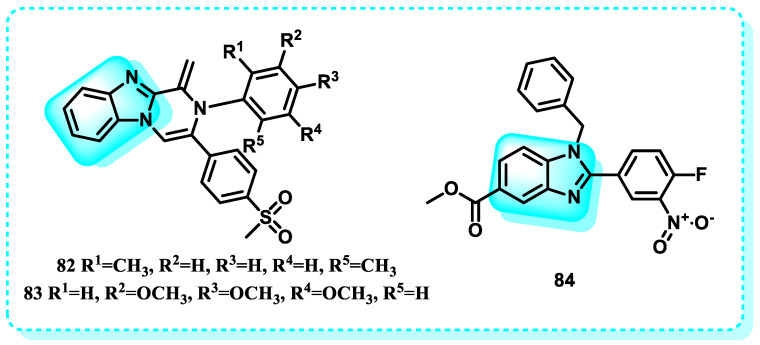
Benzimidazole derivatives that act on mitochondria.

Mitochondria function as cellular organelles responsible for energy production while also serving as the central hub for cell metabolism, controlling various crucial metabolic processes that significantly impact epigenetic regulation [239]. The metabolites that play a crucial role in DNA methylation and histone post-translational modifications, which are essential for regulating gene transcription and determining cell fate, include acetyl-CoA, α-ketoglutarate (α-KG), S-adenosyl methionine (SAM), NAD+, and O-linked beta-N-acetylglucosamine (O-GlcNAc) [240]. In this study, to ascertain the half-maximal inhibitory concentration (IC_50_), a primary culture of astrocytes derived from human glioblastoma multiforme (GBM) tissues was initially generated and exposed to compounds **82** and **83**. In the subsequent two stages, the cell death pathway and mitochondrial markers were investigated in both GBM and HEK293 cells, which are considered normal and non-cancerous (Figure 36). The Annexin/PI staining results demonstrated that **82** and **83** cause apoptosis in GBM cells, whereas the measurement of caspase indicated that apoptosis is mediated by mitochondrial signaling [241].

This research also includes the development of a novel chemical library of small benzimidazole molecules. Their potential as anticancer agents was discovered by testing them against various cancer cell lines, including Jurkat, K-562, MOLT-4, HeLa, HCT116, and MIAPaCa-2. Both MTT and trypan blue dye exclusion assays unequivocally demonstrated the cytotoxic impact of compound **84** (Figure 36). The compound can trigger programmed cell death, known as apoptosis, with a significant IC_50_ value of 1.89 ± 0.55, 2.05 ± 0.72, 3.04 ± 0.8, 3.82 ± 0.25 μM, 2.11 ± 0.62, and 1.88 ± 0.51 against K562, MOLT-4, HCT116, MIAPaCa-2, HeLa, and Jurkat cancer cell lines, respectively. Treatment with compound **84** revealed altered mitochondrial membrane potential in HeLa, HCT116, and Jurkat cells, as evidenced by JC10 labeling [242].

### 2.12. Nedd8 Activating on DNA

An essential regulator of the NEDD8 conjugation pathway is the NEDD8 activating enzyme (NAE) [243]. This route is responsible for the breakdown of many proteins with essential functions in cell cycle development, stress responses, and DNA damage [244]. This is achieved by manipulating the function of the cullin-RING subtype of ubiquitin ligases, which controls the degradation of a specific group of proteins located before the proteasome [245]. Inhibiting NAE limits the process of adding ubiquitin molecules and breaking down proteins via cullin-RING ubiquitin E3 ligases, which are involved in cancer development and progression. Multiple studies have demonstrated that the malfunction of neddylation (the process of attaching NEDD8 to target proteins) is responsible for various human illnesses, including cancer [245].

**Figure 37:**
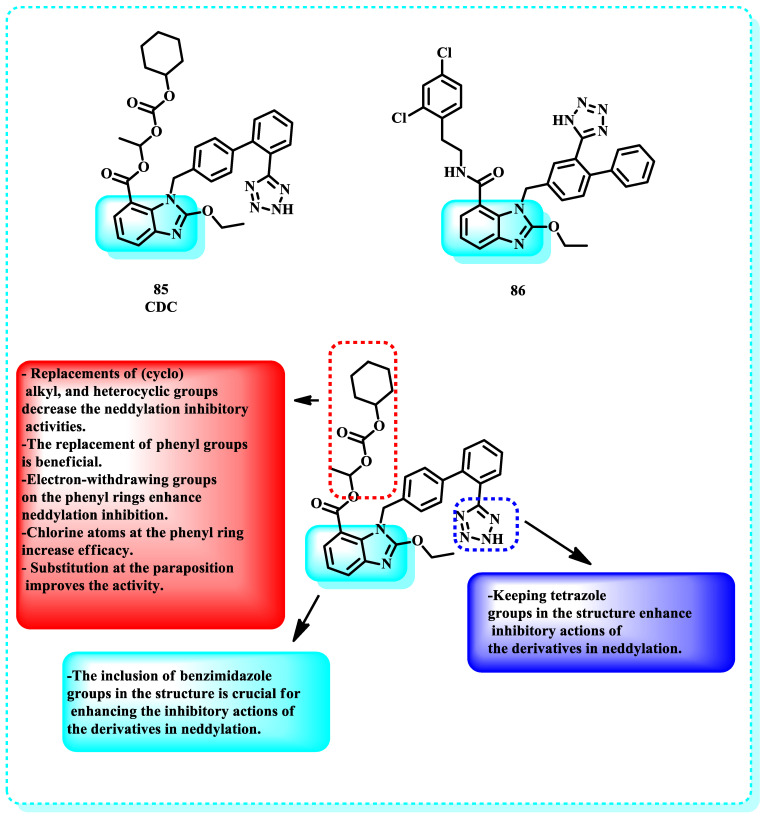
Benzimidazole derivatives acting on Nedd 8, activating on DNA, and demonstrating the structure-activity relationship of the CDC-based series.

Using candesartan cilexetil (CDC) **85** as a starting point, this research designed and synthesized 42 different benzimidazole derivatives. The purpose was to address the issue of the compound’s susceptibility to hydrolysis and enhance its ability to block neddylation, as well as its effectiveness in treating cancer. The benzimidazole-derived compound **86** exhibited enhanced neddylation inhibition in the enzyme assay compared to CDC, with an IC_50_ value of 5.51 µM versus 16.43 µM. Additionally, it demonstrated promising inhibitory efficacy against the target and selectively killed cancer cells. The findings from studying cellular mechanisms, as well as observing the inhibition of tumor growth in human lung cancer cell A549 in a living organism, along with the use of a docking model, have shown that it is possible to create a very efficient inhibitor of neddylation with compound **86** for use in cancer therapy [246]. The detailed summary of the structure-activity relationships (SARs) related to the CDC-based series is based on the structural features, early results of neddylation inhibitory abilities, and anticancer activities of the derivatives, as demonstrated in Figure 37.

### 2.13. Sirtuin Protein

Sirtuin family members possess either mono-ADP-ribosyltransferase or deacetylase activity and have a role in regulating transcriptional development in many cancer-related biological pathways [247]. Sirtuins play essential roles in cancer development and the maintenance of malignant traits in cancer cells [248]. They primarily contribute to cancer cell survival, programmed cell death, and cancer tumors. While sirtuin family members exhibit significant similarities, they may have distinct functions in different types of cancer [249].

**Figure 38:**
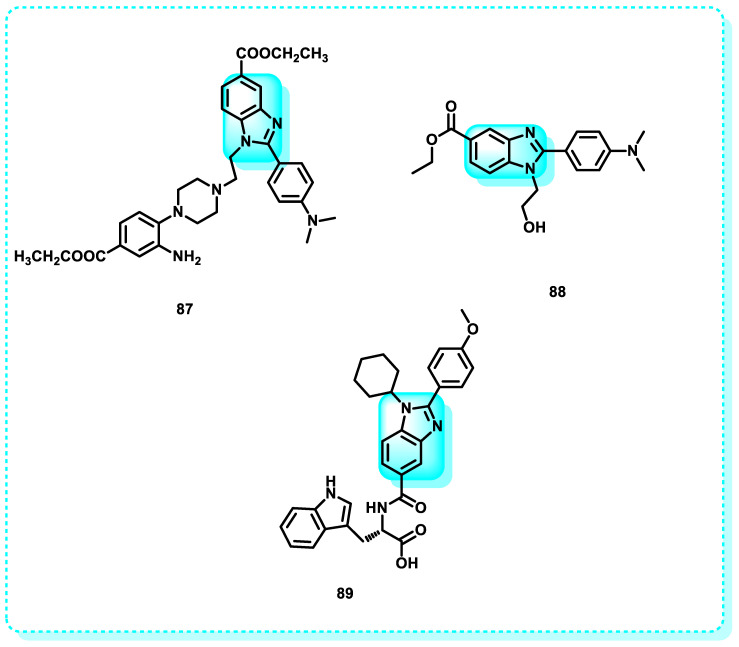
Benzimidazole derivatives that act on the Sirtuin protein.

In this study, two sets of molecules were created using the benzimidazole scaffold. The compounds were later evaluated for their SIRT1, SIRT2, and SIRT3 activity. In this investigation, three compounds exhibited significant inhibitory activity against SIRT2, with compound **87** being the most effective, displaying an IC_50_ value of 2.9 µM (Figure 38). The molecular docking analysis revealed that **87** effectively inhibited SIRT2 by displacing the cofactor NAD^+^ from its active site. A ligand-NAD^+^ competitive assay experimentally confirmed this. Compound **87** was identified as the most effective inhibitor in the series, with SIRT1 IC_50_ of 10.22 µM, SIRT2 IC_50_ of 2.92 µM, and SIRT3 IC_50_ of 10.02 µM. It is satisfying to observe that **87** exhibits greater inhibitory effects compared to AGK2, which is one of the most potent commercially accessible inhibitors of SIRT2 (Figure 38). Compound **87** is currently the most powerful sirtuin inhibitor discovered from the benzimidazole scaffold. It was discovered to be a compound that inhibits all three isoforms of SIRT1, SIRT2, and SIRT3. However, it has a greater tendency to inhibit SIRT2 specifically. The selectivity of the compound for SIRT2 is more than three times higher than its selectivity for both SIRT1 and SIRT3 [250].

Another study showed that fifteen new benzimidazole compounds were targeted, synthesized, and assessed for their ability to inhibit SIRT1 and SIRT2. All substances exhibited superior inhibition of SIRT2 in comparison to SIRT1. Out of all the compounds tested, compound **88** exhibited the highest level of inhibitory activity against SIRT1 (IC_50_ = 58.43 μM) and SIRT2 (IC50 = 45.12 μM). Compound **88** demonstrated significant anticancer activity against two distinct breast cancer cell lines (MCF-7 and MDA-MB-468), as indicated by cell cytotoxicity assays. The synthesized benzimidazole derivatives’ SAR was also demonstrated (Figure 38) [251].

Another study reveals that a set of new Sirt1 inhibitors, benzimidazole mono-peptides, was effectively designed and produced. The compounds underwent assessment for their inhibitory effects on the enzyme Sirt1 by in vitro enzyme-based and cell-based assays. Additionally, their cytotoxic activity was tested in liver and breast cancer cells. The compound **89** exhibited the highest efficiency in inhibiting Sirt1, with an IC_50_ value of 0.77 μM and a ΔG_bind_ value of −4.4 kcal mol^−1^ [242].

### 2.14. Peptidyl Prolyl Isomerase

PPIases are present in all living creatures. Three families of PPIases, namely cyclophilin (CyP), FK506 binding protein (FKBP), and parvulin (Pvn), have been identified [252]. PPIases facilitate the cis/trans isomerization of proline, serving as a regulatory mechanism throughout the folding, activation, and degradation of several proteins. These “clients” encompass proteins that play crucial roles in cancer, neurodegeneration, and psychiatric diseases, indicating that PPIase inhibitors may hold significant therapeutic value [253].

**Figure 39:**
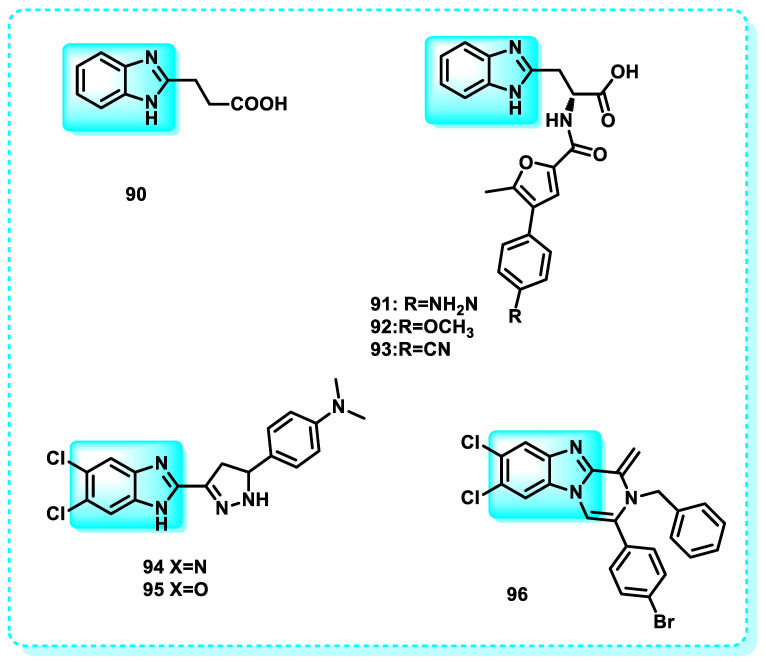
Benzimidazole derivatives that act on peptidyl prolyl isomerase.

This paper presents the discovery and expansion of a benzimidazole series. Based on a structural analysis technique and analog screening, compound **90** was discovered with nanomolar efficacy against Pin1. Compound **90** crystallized in the active site of the PPIase K77Q/K82Q variant of Pin1. The ligand efficiency of compound **90** (LE = 0.29) was deemed satisfactory to hit it as a starting point. Compounds **91**–**93** showed bitter activity with Pin1 IC_50_ (µM) = 0.025, 0.13, and 0.18, respectively (Figure 39) [254].

Another study in which a group of benzimidazole derivatives was synthesized to target breast cancer. The synthesis of benzimidazole scaffolds, which were conjugated with pyrazanobenzimidazoles and pyridobenzimidazole linkers, was carried out. Compounds **94**, **95**, and **96**, which exhibit the most potent anti-breast cancer action against the MCF-7 cell line with IC_50_ = 11.7 ± 0.9, 8.7 ± 0.6, and 31.3 ± 2.5 µM, respectively, were chosen for the Pin1 inhibition experiment (Figure 39). Tannic acid was used as a reference pharmacological control. The impact of Compound **95** on apoptosis and its potential to induce apoptosis were investigated [255].

### 2.15. F-Actin

Filamentous actin (F-actin) is a crucial protein that forms the structural framework of the cytoskeleton in cells. It is included in different cellular processes, such as division and movement of the cells and the transport of vesicles [256]. The set consists of six distinct isoforms relevant to different cell types: ACTA1-2, ACTB, ACTC1, ACTG1, and ACTG2. Aberrant expression of actin isoforms has been observed in numerous types of cancer, prompting us to propose that it could potentially function as an early indicator of cancer [257].

**Figure 40:**
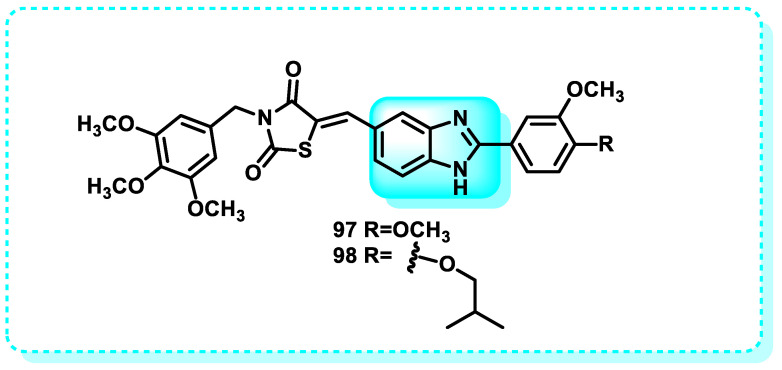
Benzimidazole derivatives that act on F-actin.

A set of benzimidazole-based thiazolidinedione derivatives was designed and synthesized. Among these compounds **97** and **98** exhibited potential cytotoxicity against PC-3, HeLa, A549, and HT1080 cancer cells, with IC_50_ values ranging from 0.096 to 0.63 µM. Treatment with **97** and **98** induced typical apoptotic changes in the cells (Figure 40). Additionally, these compounds inhibited cell migration by disrupting the assembly of the F-actin protein [258].

## 3. Conclusions and Future Perspectives

Benzimidazole remains a crucial scaffold in contemporary medicinal chemistry due to its structural versatility and proven effectiveness in various therapeutic domains. The application of structure–activity relationship insights, combined with advanced computational modeling, will expedite the identification of next-generation benzimidazole-based agents with enhanced potency, selectivity, and safety profiles. These innovations hold promise in tackling global health challenges, including antimicrobial resistance, cancer treatment limitations, and viral infections.

This review categorizes benzimidazoles as anticancer agents based on diverse biological properties, aiming to support the rational design of the novel derivatives by analyzing SAR and therapeutic potential. Notably, several benzimidazole-based drugs have achieved clinical authorization, highlighting the scaffold’s promise in oncology. Benzimidazole hybrids can augment efficacy, surmount multidrug resistance, mitigate adverse effects, and enhance pharmacokinetics, pharmacodynamics, and physicochemical characteristics. Their ability to target multiple cancer pathways without drug–drug interactions is particularly encouraging for multi-targeted therapies.

However, despite these advances, several limitations remain. Challenges include suboptimal solubility and bioavailability, potential off-target effects, and incomplete understanding of resistance mechanisms. Furthermore, the complexity of cancer biology demands more extensive in vivo and clinical validation of lead compounds. Future research should focus on overcoming these limitations through innovative drug delivery systems, further exploration of the molecular mechanisms underlying activity, resistance, and expanding the chemical diversity of benzimidazole hybrids with novel heterocycles and substituents. Integration of artificial intelligence and machine learning in drug design may also accelerate optimization processes.

In summary, while benzimidazole derivatives exhibit significant anticancer potential, addressing current challenges and focusing on mechanistic insights and translational studies are essential to fully harness their therapeutic capabilities. This review aims to stimulate ongoing multidisciplinary efforts toward developing safer and more effective benzimidazole-based cancer therapeutics.

## Figures and Tables

**Table 1:** Pharmacological effects of drugs that contain the *1H*-benzimidazole moiety.

CLASS	Structure and Name of the Drug		
Anticancer	** 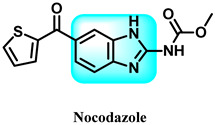 **	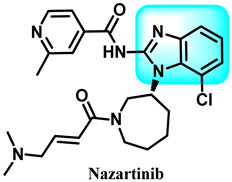	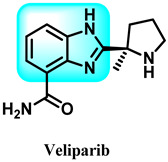
	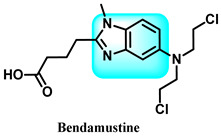	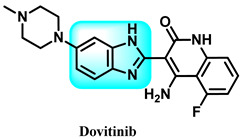	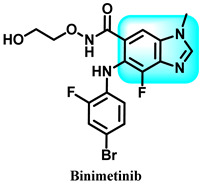
	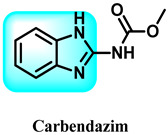	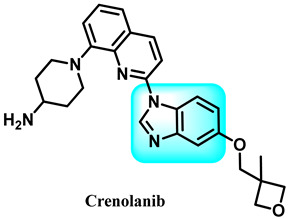	
Antihistamine	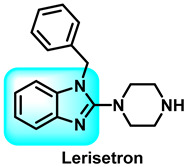	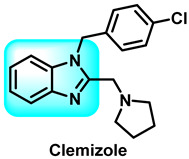	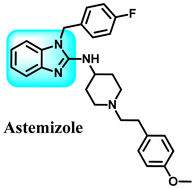
	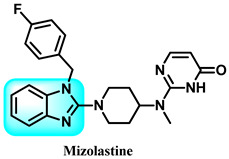	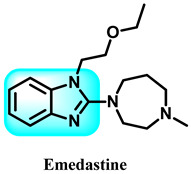	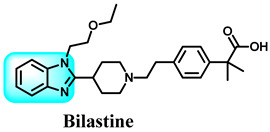
Antiparasitic, Anthelmintic	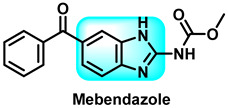	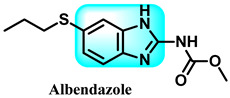	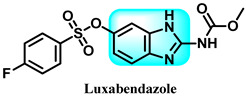
	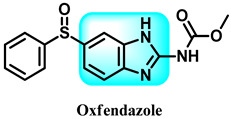	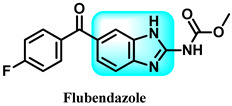	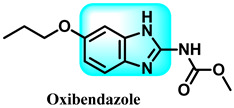
	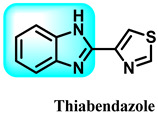	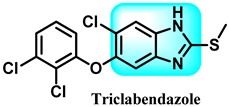	
Antihypertensive	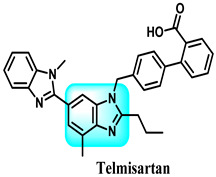	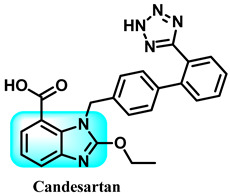	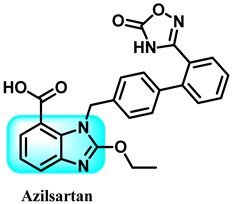
Antiviral	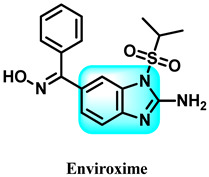	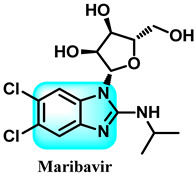	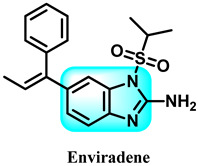
	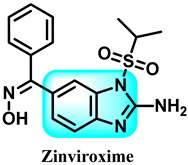		
Antifungal	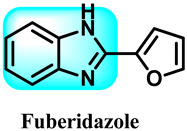	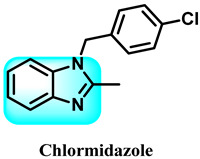	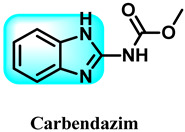
	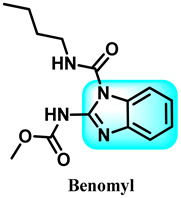		
PPI	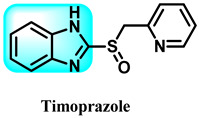	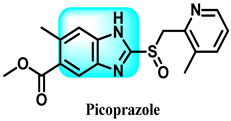	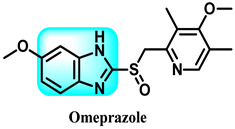
	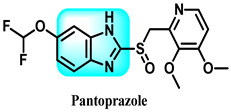	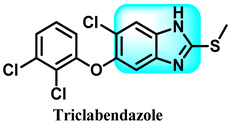	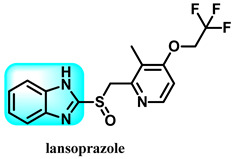
	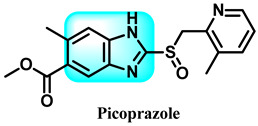		
Analgesic	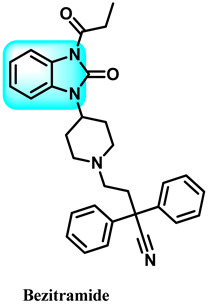		
Antiallergic	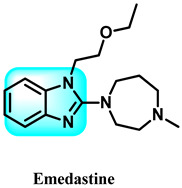		
Antiemetic	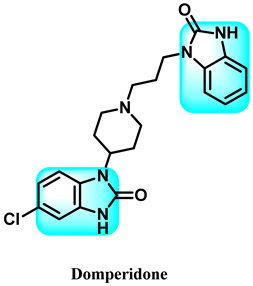		
uricosuric	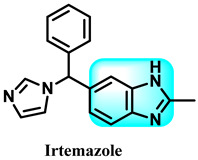		
Psychopharmacological agent	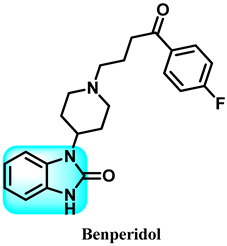		
Anti-dopaminergic, antiemetic, and antipsychotic	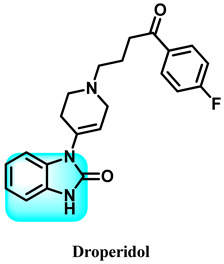		
Cardiotonic agent	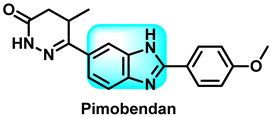		

**Table 2:** IC_50_ cytotoxicity data of tested molecules **1** and **2** in μM.

Cell Line	Compound 1	Compound 2	Etoposide
MCF-7	0.092 ± 0.001	0.81 ± 0.062	2.11 ± 0.024
A549	0.72 ± 0.042	1.90 ± 0.88	3.08 ± 0.135
Colo-205	0.34 ± 0.071	0.41 ± 0.12	0.13 ± 0.017
A2780	1.23 ± 0.55	1.80 ± 0.59	1.31 ± 0.27

**Table 3:** Demonstrations of the binding energies of compounds **1** and **2** to the active amino acids.

Compounds	Compound 1		Compound 2	
	Binding Energy	Active Site Residues	Binding Energy	Active Site Residues
2J5F	−5.22	Arg841, Asn842, Asp855	−5.17	Arg841, Asp855
5 × 8I	−7.35	Phe241, Lys290	−8.52	Phe241
6FYL	−6.69	Leu246	−7.76	Leu246
2WU6	−7.33	Lys241, Leu239	−8.04	Leu239
6FYV	−5.52	Leu244, Lys191	−5.42	None
5UL1	−7.84	Trp850	−5.69	Trp850
5IZA	−8.15	None	−7.44	Lys603, Arg649

**Table 4:** Data on Chk2 binding for 2-arylbenzimidazole derivatives.

Z	R	IC_50_ (µM)
-S-	4-OH	2.3 ± 0.5
-SO_2_NH-	H	18 ± 0.8
-SO_2_NH-	3,4-diCl	3.4 ± 1.3
-O-	H	55 ± 31
-O-	3,4-diCl	5.5 ± 1.1
-CONH-	H	410 ± 70
-S-	4-Cl	8.2 ± 2.0
-CONH-	4-Cl	680 ± 13
-SO_2_NH-	4-Cl	14 ± 4.1
-CONH-	3,4-diCl	>10,000
-O-	4-Cl	16 ± 6.9

**Table 5:** Affinity for binding to receptors for benzimidazole series (**16**–**19**).

Eraselectivity x-Fold Compounds	TR-FRET Erα IC_50_ (nM)	RBA-Erα (%)	TR-FRET ER β IC_50_ (nM)	RBA-ERβ (%)	Erα Selectivity x-Fold
**16**	6.20 ± 1.52	7.52	7.89 ± 2.04	6.78	1.11
**17**	11.52 ± 1.53	4.04	6.09 ± 2.20	8.79	0.46
**18**	7.56 ± 1.57	6.17	6.69 ± 1.24	8.00	0.77
**19**	0.75 ± 0.94	6.91	10.8 ± 3.50	4.96	1.39

## Data Availability

No new data were created or analyzed in this study.
